# Application of Reinforcement Learning in Cognitive Radio Networks: Models and Algorithms

**DOI:** 10.1155/2014/209810

**Published:** 2014-06-05

**Authors:** Kok-Lim Alvin Yau, Geong-Sen Poh, Su Fong Chien, Hasan A. A. Al-Rawi

**Affiliations:** ^1^Faculty of Science and Technology, Sunway University, No. 5 Jalan Universiti, Bandar Sunway, 46150 Petaling Jaya, Selangor, Malaysia; ^2^University Malaysia of Computer Science & Engineering, Jalan Alamanda 2, Presint 16, 62150 Putrajaya, Wilayah Persekutuan Putrajaya, Malaysia; ^3^Department of Mathematical Modeling Laboratory, Mimos Berhad, Technology Park Malaysia, 57000 Kuala Lumpur, Malaysia

## Abstract

Cognitive radio (CR) enables unlicensed users to exploit the underutilized spectrum in licensed spectrum whilst minimizing interference to licensed users. Reinforcement learning (RL), which is an artificial intelligence approach, has been applied to enable each unlicensed user to observe and carry out optimal actions for performance enhancement in a wide range of schemes in CR, such as dynamic channel selection and channel sensing. This paper presents new discussions of RL in the context of CR networks. It provides an extensive review on how most schemes have been approached using the traditional and enhanced RL algorithms through state, action, and reward representations. Examples of the enhancements on RL, which do not appear in the traditional RL approach, are rules and cooperative learning. This paper also reviews performance enhancements brought about by the RL algorithms and open issues. This paper aims to establish a foundation in order to spark new research interests in this area. Our discussion has been presented in a tutorial manner so that it is comprehensive to readers outside the specialty of RL and CR.

## 1. Introduction

Cognitive radio (CR) [1] is the next generation wireless communication system that enables unlicensed or Secondary Users (SUs) to explore and use underutilized licensed spectrum (or white spaces) owned by the licensed or Primary Users (PUs) in order to improve the overall spectrum utilization. The CR technology improves the availability of bandwidth at each SU, and so it enhances the SU network performance. Reinforcement learning (RL) has been applied in CR so that the SUs can observe, learn, and take optimal actions on their respective local operating environment. For example, a SU observes its spectrum to identify white spaces, learns the best possible channels for data transmissions, and takes actions such as to transmit data in the best possible channel. Examples of schemes in which RL has been applied are dynamic channel selection [2], channel sensing [3], and routing [4]. To the best of our knowledge, the discussion on the application of RL in CR networks is new albeit the importance of RL in achieving the fundamental concept of CR, namely, cognition cycle (see Section 2.2.1). This paper provides an extensive review on various aspects of the application of RL in CR networks, particularly, the components, features, and enhancements of RL. Most importantly, we present how the traditional and enhanced RL algorithms have been applied to approach most schemes in CR networks. Specifically, for each new RL model and algorithm which is our focus, we present the purpose(s) of a CR scheme, followed by in-depth discussion on its associated RL model (i.e., state, action, and reward representations) which characterizes the purposes, and finally the RL algorithm which aims to achieve the purpose. Hence, this paper serves as a solid foundation for further research in this area, particularly, for the enhancement of RL in various schemes in the context of CR, which can be achieved using new extensions in existing schemes, and for the application of RL in new schemes.

The rest of this paper is organized as follows.  Section 2 presents RL and CR networks.  Section 3 presents various components, features, and enhancements of RL in the context of CR networks.  Section 4 presents various RL algorithms in the context of CR networks.  Section 5 presents performance enhancements brought about by the RL algorithms in various schemes in CR networks.  Section 6 presents open issues.  Section 7 presents conclusions.

## 2. Reinforcement Learning and Cognitive Radio Networks

This section presents an overview of RL and CR networks.

### 2.1. Reinforcement Learning

Reinforcement learning is an unsupervised and online artificial intelligence technique that improves system performance using simple modeling [5]. Through unsupervised learning, there is no external teacher or critic to oversee the learning process, and so, an agent learns knowledge about the operating environment by itself. Through online learning, an agent learns knowledge on the fly while carrying out its normal operation, rather than using empirical data or experimental results from the laboratory.

**Figure 1:**
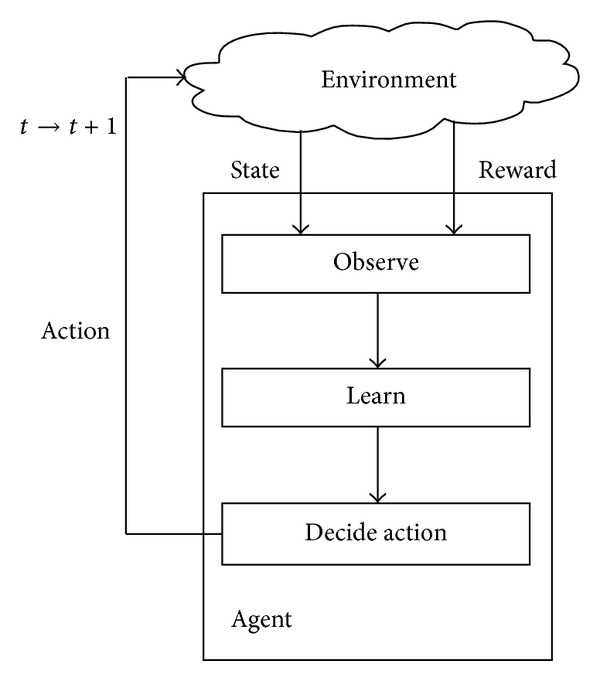
A simplified RL model.

Figure 1 shows a simplified version of a RL model. At a particular time instant, a learning agent or a decision maker observes state and reward from its operating environment, learns, decides, and carries out its action. The important representations in the RL model for an agent are as follows.

- * State* represents the decision-making factors, which affect the reward (or network performance), observed by an agent from the operating environment. Examples of states are the channel utilization level by PUs and channel quality.
- * Action* represents an agent's action, which may change or affect the state (or operating environment) and reward (or network performance), and so the agent learns to take optimal actions at most of the times.
- * Reward* represents the positive or negative effects of an agent's action on its operating environment in the previous time instant. In other words, it is the consequence of the previous action on the operating environment in the form of network performance (e.g., throughput).

At any time instant, an agent observes its state and carries out a proper action so that the state and reward, which are the consequences of the action, improve in the next time instant. Generally speaking, RL estimates the reward of each state-action pair, and this constitutes knowledge. The most important component in Figure 1 is the learning engine that provides knowledge to the agent. We briefly describe how an agent learns. At any time instant, an agent's action may affect the state and reward for better or for worse or maintain the status quo; and this in turn affects the agent's next choice of action. As time progresses, the agent learns to carry out a proper action given a particular state. As an example of the application of the RL model in CR networks, the learning mechanism is used to learn channel conditions in a dynamic channel selection scheme. The state represents the channel utilization level by PUs and channel quality. The action represents a channel selection. Based on an application, the reward represents distinctive performance metrics such as throughput and successful data packet transmission rate. Lower channel utilization level by PUs and higher channel quality indicate better communication link, and hence the agent may achieve better throughput performance (reward). Therefore, maximizing reward provides network performance enhancement.

*Q*-learning [5] is a popular technique in RL, and it has been applied in CR networks. Denote decision epochs by *t* ∈ *T* = {1,2,…}; the knowledge possessed by agent *i* for a particular state-action pair at time *t* is represented by *Q*-function as follows:

(1) Qt+1i(sti,ati)⟵(1−α)Qti(sti,ati) +α[rt+1i(st+1i)+γmax⁡a∈A⁡Qti(st+1i,a)],

where

- *s*_*t*_^*i*^ ∈ *S* represents state,
- *a*_*t*_^*i*^ ∈ *A* represents action,
- *r*_*t*+1_^*i*^(*s*_*t*+1_^*i*^) ∈ *R* represents delayed rewards, which is received at time *t* + 1 for an action taken at time *t*,
- 0 ≤ *γ* ≤ 1 represents discount factor. The higher the value of *γ*, the greater the agent relies on the discounted future reward *γ*max⁡_*a*∈*A*_⁡*Q*_*t*_^*i*^(*s*_*t*+1_^*i*^, *a*) compared to the delayed reward *r*_*t*+1_^*i*^(*s*_*t*+1_^*i*^),
- 0 ≤ *α* ≤ 1  represents learning rate. The higher the value of *α*, the greater the agent relies on the delayed reward *r*_*t*+1_^*i*^(*s*_*t*+1_^*i*^) and the discounted future reward *γ*max⁡_*a*∈*A*_⁡*Q*_*t*_^*i*^(*s*_*t*+1_^*i*^, *a*), compared to the *Q*-value *Q*_*t*_^*i*^(*s*_*t*_^*i*^, *a*_*t*_^*i*^) at time *t*.

At decision epoch *t*, agent *i* observes its operating environment to determine its current state *s*_*t*_^*i*^. Based on the *s*_*t*_^*i*^, the agent chooses an action *a*_*t*_^*i*^. Next, at decision epoch *t* + 1, the state *s*_*t*_^*i*^  changes to *s*_*t*+1_^*i*^ as a consequence of the action *a*_*t*_^*i*^, and the agent receives delayed reward *r*_*t*+1_^*i*^(*s*_*t*+1_^*i*^). Subsequently, the *Q*-value *Q*_*t*+1_^*i*^(*s*_*t*_^*i*^, *a*_*t*_^*i*^) is updated using (1). Note that, in the remaining decision epochs at time *t*, *t* + 1,…, the agent is expected to take optimal actions with regard to the states; hence, *Q*-value is updated using a maximized discounted future reward *γ*max⁡_*a*∈*A*_⁡*Q*_*t*_^*i*^(*s*_*t*+1_^*i*^, *a*). As this procedure evolves through time, agent *i* receives a sequence of rewards and the *Q*-value converges. Q-learning searches for an optimal policy at all time instants through maximizing value function *V* ^*π*^(*s*_*t*_^*i*^) as shown below:

(2) $$\begin{array}{c} V^{\pi }\left(s_{t}^{i}\right)=\underset{a\in A}{\max}\left(Q_{t}^{i}\left(s_{t}^{i},a\right)\right). \end{array}$$

Hence, the policy (or action selection) for agent *i* is as follows:

(3) $$\begin{array}{c} \pi_{i}\left(s_{t}^{i}\right)=\arg\underset{a\in A}{\max}\left(Q_{t}^{i}\left(s_{t}^{i},a\right)\right). \end{array}$$

The update of the *Q*-value in (1) does not cater for the actions that are never chosen. Exploitation chooses the best-known action, or the greedy action, at all time instants for performance enhancement. Exploration chooses the other nonoptimal actions once in a while to improve the estimates of all *Q*-value in order to discover better actions. While Figure 1 shows a single agent, the presence of multiple agents is feasible. In the context of CR networks, a rigorous proof of the convergence of *Q*-value in the presence of multiple SUs has been shown in [6].

The advantages of RL are as follows:

- instead of tackling every single factor that affects the system performance, RL models the system performance (e.g., throughput) that covers a wide range of factors affecting the throughput performance including the channel utilization level by PUs and channel quality and, hence, its simple modeling approach;
- prior knowledge of the operating environment is not necessary; and so a SU can learn the operating environment (e.g., channel quality) as time goes by.

### 2.2. Cognitive Radio Networks

Traditionally, spectrum allocation policy has been partitioning radio spectrum into smaller ranges of licensed and unlicensed frequency bands (also called channels). The licensed channels provide exclusive channel access to licensed users or PUs. Unlicensed users or SUs, such as the popular wireless communication systems IEEE 802.11, access unlicensed channels without incurring any monetary cost, and they are forbidden to access any of the licensed channels. Examples of unlicensed channels are Industrial, Scientific, and Medical (ISM) and Unlicensed National Information Infrastructure (UNII) bands. While the licensed channels have been underutilized, the opposite phenomenon has been observed among the unlicensed channels.

**Figure 2:**
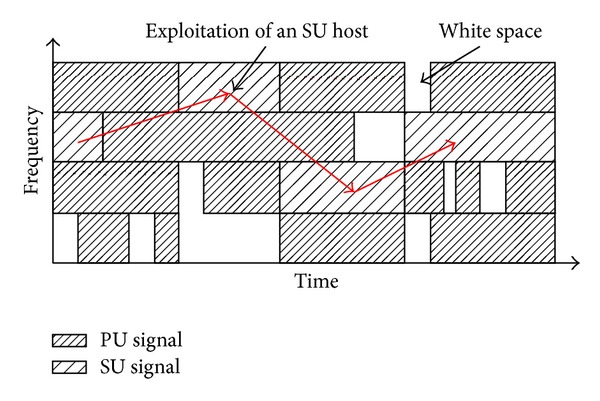
A SU exploits white spaces across various channels.

Cognitive radio enables SUs to explore radio spectrum and use white spaces whilst minimizing interference to PUs. The purpose is to improve the availability of bandwidth at each SU, hence improving the overall utilization of radio spectrum. CR helps the SUs to establish a “friendly” environment, in which the PUs and SUs coexist without causing interference with each other as shown in Figure 2. In Figure 2, a SU switches its operating channel across various channels from time to time in order to utilize white spaces in the licensed channels. Note that each SU may observe different white spaces, which are location dependent. The SUs must sense the channels and detect the PUs' activities whenever they reappear in white spaces. Subsequently, the SUs must vacate and switch their respective operating channel immediately in order to minimize interference to PUs. For a successful communication, a particular white space must be available at both SUs in a communication node pair.

The rest of this subsection is organized as follows. Section 2.2.1 presents cognition cycle, which is an essential component in CR.  Section 2.2.2 represents various application schemes in which RL has been applied to provide performance enhancement.

#### 2.2.1. Cognition Cycle

Cognition cycle [7], which is a well-known concept in CR, is embedded in each SU to achieve context awareness and intelligence in CR networks. Context awareness enables a SU to sense and be aware of its operating environment; while intelligence enables the SU to observe, learn, and use the white spaces opportunistically so that a static predefined policy is not required while providing network performance enhancement.

The cognition cycle can be represented by a RL model as shown in Figure 1. The RL model can be tailored to fit well with a wide range of applications in CR networks. A SU can be modeled as a learning agent. At a particular time instant, the SU agent observes state and reward from its operating environment, learns, decides, and carries out action on the operating environment in order to maximize network performance. Further description on RL-based cognition cycle is presented in Section 2.1.

#### 2.2.2. Application Schemes

Reinforcement learning has been applied in a wide range of schemes in CR networks for SU performance enhancements, whilst minimizing interference to PUs. The schemes are listed as follows, and the nomenclatures (e.g., (A1) and (A2)) are used to represent the respective application schemes throughout the paper.

(A1) * Dynamic Channel Selection (DCS).* The DCS scheme selects operating channel(s) with white spaces for data transmission whilst minimizing interference to PUs. Yau et al. [8, 9] propose a DCS scheme that enables SUs to learn and select channels with low packet error rate and low level of channel utilization by PUs in order to enhance QoS, particularly throughput and delay performances.
(A2) * Channel Sensing.* Channel sensing senses for white spaces and detects the presence of PU activities. In [10], the SU reduces the number of sensing channels and may even turn off channel sensing function if its operating channel has achieved the required successful transmission rate in order to enhance throughput performance. In [11], the SU determines the durations of channel sensing, time of channel switching, and data transmission, respectively, in order to enhance QoS, particularly throughput, delay, and packet delivery rate performances. Both [10, 11] incorporate DCS (A1) into channel sensing in order to select operating channels. Due to the environmental factors that can deteriorate transmissions (e.g., multipath fading and shadowing), Lo and Akyildiz [3] propose a cooperative channel sensing scheme, which combines sensing outcomes from cooperating one-hop SUs, to improve the accuracy of PU detection.
(A3) * Security Enhancement.* Security enhancement scheme [12] aims to ameliorate the effects of attacks from malicious SUs. Vucevic et al. [13] propose a security enhancement scheme to minimize the inaccurate sensing outcomes received from neighboring SUs in channel sensing (A2). A SU becomes malicious whenever it sends inaccurate sensing outcomes, intentionally (e.g., Byzantine attacks) or unintentionally (e.g., unreliable devices). Wang et al. [14] propose an antijamming scheme to minimize the effects of jamming attacks from malicious SUs, which constantly transmit packets to keep the channels busy at all times so that SUs are deprived of any opportunities to transmit.
(A4) * Energy Efficiency Enhancement.* Energy efficiency enhancement scheme aims to minimize energy consumption. Zheng and Li [15] propose an energy-efficient channel sensing scheme to minimize energy consumption in channel sensing. Energy consumption varies with activities, and it increases from sleep, idle, to channel sensing. The scheme takes into account the PU and SU traffic patterns and determines whether a SU should enter sleep, idle, or channel sensing modes. Switching between modes should be minimized because each transition between modes incurs time delays.
(A5) * Channel Auction.* Channel auction provides a bidding platform for SUs to compete for white spaces. Chen and Qiu [16] propose a channel auction scheme that enables the SUs to learn the policy (or action selection) of their respective SU competitors and place bids for white spaces. This helps to allocate white spaces among the SUs efficiently and fairly.
(A6) * Medium Access Control (MAC).* MAC protocol aims to minimize packet collision and maximize channel utilization in CR networks. Li et al. [17] propose a collision reduction scheme that reduces the probability of packet collision among PUs and SUs, and it has been shown to increase throughput and to decrease packet loss rate among the SUs. Li et al. [18] propose a retransmission policy that enables a SU to determine how long it should wait before transmission in order to minimize channel contention.
(A7) * Routing.* Routing enables each SU source or intermediate node to select its next hop for transmission in order to search for the best route(s), which normally incurs the least cost or provides the highest amount of rewards, to the SU destination node. Each link within a route has different types and levels of costs, such as queuing delay, available bandwidth or congestion level, packet loss rate, energy consumption level, and link reliability, as well as changes in network topology as a result of irregular node's movement speed and direction.
(A8) * Power Control.* Yao and Feng [19] propose a power selection scheme that selects an available channel and a power level for data transmission. The purpose is to improve its Signal-to-Noise Ratio (SNR) in order to improve packet delivery rate.

## 3. Reinforcement Learning in the Context of Cognitive Radio Networks: Components, Features, and Enhancements

This section presents the components of RL, namely, state, action, reward, discounted reward, and *Q*-function; as well as the features of RL, namely, exploration and exploitation, updates of learning rate, rules and cooperative learning. The components and features of RL (see Section 2.1) are presented in the context of CR. For each component and feature, we show the traditional approach and subsequently the alternative or enhanced approaches with regard to modeling, representing, and applying them in CR networks. This section serves as a foundation for further research in this area, particularly, the application of existing features and enhancements in current schemes in RL models for either existing or new schemes.

Note that, for improved readability, the notations (e.g., *s*_*t*_^*i*^ and *a*_*t*_^*i*^) used in this paper represent the same meaning throughout the entire paper, although different references in the literature may use different notations for the same purpose.

### 3.1. State

Traditionally, each state is comprised of a single type of information. For instance, in [11], each state *s*_*t*_^*i*^ ∈ *S* = {1,2,…, *K*} represents a single channel out of *K* channels available for data transmission. The state may be omitted in some cases. For instance, in [10], the state and action representations are similar, so the state is not represented. The traditional state representation can be enhanced in the context of CR as described next.

Each state can be comprised of several types of information. For instance, Yao and Feng [19] propose a joint DCS (A1) and power allocation (A8) scheme in which each state is comprised of three-tuple information; specifically, **s**_**t**_^**i**^ = (*s*_1,*t*_^*i*^, *s*_2,*t*_^*i*^, *s*_3,*t*_^*i*^) ∈ *S*_1_ × *S*_2_ × *S*_3_. The substate *s*_1,*t*_^*i*^ ∈ *S*_1_ = {1,2,…, *N*_SU_} represents the number of SU agents, *s*_2,*t*_^*i*^ ∈ *S*_2_ = {1,2,…, *N*_SU-SU_} represents the number of communicating SU agents, and *s*_3,*t*_^*i*^ ∈ *S*_3_ = {*p*_1_, *p*_2_,…, *p*_*N*_*rp*__} represents the received power on each channel.

The value of a state may deteriorate as time goes by. For instance, Lundén et al. [20] propose a channel sensing (A2) scheme in which each state *s*_*k*,*t*_^*i*^ ∈ {0 ≤ *p*_idle,*k*_^*i*^ ≤ 1} represents SU agent *i*'s belief (or probability) that channel *k* is idle (or the absence of PU activity). Note that the belief value of channel *k* deteriorates whenever the channel is not sensed recently, and this indicates the diminishing confidence in the belief that channel *k* remains idle. Denote a small step size by *δ* (i.e., *δ* = 0.01); the state value of channel *k* deteriorates if it is not updated at each time instant; specifically, *s*_*k*,*t*+1_^*i*^ = *s*_*k*,*t*_^*i*^ − *δ*.

### 3.2. Action

Traditionally, each action represents a single action *a*_*t*_^*i*^ out of a set of possible actions *A*. For instance, in [10], each action *a*_*t*_^*i*^ ∈ *A* = {1,2,…, *K*} represents a single channel out of the *K* channels available for data transmission. The traditional action representation can be enhanced in the context of CR as described next.

Each action *a*_*t*_^*i*^ ∈ *A* can be further divided into various levels. As an example, Yao and Feng [19] propose a joint DCS (A1) and power allocation (A8) scheme in which each action *a*_*t*_^*i*^ ∈ *A* = {*p*_1_, *p*_2_,…, *p*_*K*_} represents a channel selection, and each *p*_*k*_ ∈ *P*_PA_ = {*p*_1_, *p*_2_,…, *p*_*N*_PA__} represents a power level allocation with *N*_PA_ being the number of power levels. As another example, Zheng and Li [15] propose an energy efficiency enhancement (A4) scheme in which there are four kinds of actions, namely, transmit, idle, sleep, and sense channel. The sleepaction *a*_sp,*t*_^*i*^ ∈ *A* = {*a*_sp1_, *a*_sp2_,…, *a*_*N*_sp__} represents a sleep level with *N*_sp_ being the number of sleep levels. Note that different sleep level incurs different amount of energy consumption.

### 3.3. Delayed Reward

Traditionally, each delayed reward represents the amount of performance enhancement achieved by a state-action pair. A single reward computation approach is applicable to all state-action pairs. As an example, in [2], *r*_*t*+1_^*i*^(*a*_*t*+1_^*i*^) ∈ *R* = {1, −1} represents the reward and cost values of 1 and −1 for each successful and unsuccessful transmission, respectively. As another example, in [8], *r*_*t*+1_^*i*^(*a*_*t*+1_^*i*^) represents the amount of throughput achieved within a time window. The traditional reward representation can be enhanced in the context of CR as described next.

The delayed reward can be computed differently for distinctive actions. As an example, in a joint DCS (A1) and channel sensing (A2) scheme, Felice et al. [21] compute the delayed rewards in two different ways based on the types of actions: channel sensing *a*_se_ and data transmission *a*_tx_. Firstly, a SU agent calculates delayed reward *r*_*t*+1_^*i*^(*s*_*t*_^*i*^, *a*_se,*t*_^*i*^) at time instant *t* + 1. The *r*_*t*+1_^*i*^(*s*_*t*_^*i*^, *a*_se,*t*_^*i*^) indicates the likelihood of the existence of PU activities in channel *s*_*t*_^*i*^ whenever action *a*_se,*t*_^*i*^ is taken. Specifically, *r*_*t*+1_^*i*^(*s*_*t*_^*i*^, *a*_se,*t*_^*i*^) = ∑_*j*=0_^*N*_nbr,*i*_^ *d*_*i*,*j*_/*N*_nbr,*i*_ where *N*_nbr,*i*_ indicates the number of neighboring SU agents, while *d*_*i*,*j*_, which is a binary value, indicates the existence of PU activities as reported by SU neighbor agent *j* ∈ *N*_nbr,*i*_. Secondly, a SU agent calculates delayed reward *r*_*t*+1_^*i*^(*s*_*t*_^*i*^, *a*_tx,*t*_^*i*^) at time instant *t* + 1. The *r*_*t*+1_^*i*^(*s*_*t*_^*i*^, *a*_tx,*t*_^*i*^) indicates the successful transmission rate, which takes into account the aggregated effect of interference from PU activities whenever action *a*_tx,*t*_^*i*^ is taken. Specifically, *r*_*t*+1_^*i*^(*s*_*t*_^*i*^, *a*_tx,*t*_^*i*^) = ∑_*j*=0_^*N*_DATA,*i*_^ACK_*i*,*j*_/∑_*j*=0_^*N*_DATA,*i*_^DATA_*i*,*j*_ where *N*_DATA,*i*_ indicates the number of data packets sent by SU agent *i*, ACK_*i*,*j*_ indicates the number of acknowledgment packets received by SU agent *i*, and DATA_*i*,*j*_ indicates the number of data packets being transmitted by SU agent *i*.

Jouini et al. [22] apply an Upper Confidence Bound (UCB) algorithm to compute delayed rewards in a dynamic and uncertain operating environment (e.g., operating environment with inaccurate sensing outcomes), and it has been shown to improve throughput performance in DCS (A1). The main objective of this algorithm is to determine the upper confidence bounds for all rewards and subsequently use them to make decisions on action selection. The rewards are uncertain, and the uncertainty is caused by the dynamicity and uncertainty of the operating environment. Let *N*_*a*^*i*^_(*t*) represent the number of times an action *a* ^*i*^ ∈ *A* has been taken on the operating environment up to time *t*; an agent *i* calculates the upper confidence bounds of all delayed rewards as follows:

(4) $$\begin{array}{c} B_{t}^{i}\left(a_{t}^{i},N_{a^{i}}\left(t\right)\right)=\overset{-}{r_{t}^{i}}\left(a_{t}^{i},N_{a^{i}}\left(t\right)\right)+U_{t}^{i}\left(a_{t}^{i},N_{a^{i}}\left(t\right)\right), \end{array}$$

where $\overset{-}{r_{t}^{i}}\left(a_{t}^{i},N_{a^{i}}(t)\right)=\sum_{j=0}^{t-1}r_{j}^{i}(a_{j}^{i})/N_{a^{i}}(t)$ is the mean reward, and *U*_*t*_(*a*_*t*_^*i*^, *N*_*a*^*i*^_(*t*)) is the upper confidence bias being added to the mean. Note that *r*_*j*_^*i*^(*a*_*j*_^*i*^) = 0 if *a*_*j*_^*i*^ is not chosen at time instant *j*. The *U*_*t*_^*i*^(*a*_*t*_^*i*^, *N*_*a*^*i*^_(*t*)) is calculated as follows:

(5) $$\begin{array}{c} U_{t}^{i}\left(a_{t}^{i},N_{a^{i}}\left(t\right)\right)=\sqrt{\frac{\beta \cdot \ln\left(t\right)}{N_{a^{i}}\left(t\right)}}, \end{array}$$

where exploration coefficient *β* > 1 is a constant empirical factor. For instance, *β* = 1.2 in [22, 23].

The UCB algorithm selects actions with the highest upper confidence bounds, and so (3) is rewritten as follows:

(6) $$\begin{array}{c} \pi_{i}\left(a_{t}^{i}\right)=\arg\underset{a\in A}{\max}B_{t}^{i}\left(a,N_{a^{i}}\left(t\right)\right). \end{array}$$

### 3.4. Discounted Reward

Traditionally, the discounted reward has been applied to indicate the dependency of *Q*-value on future rewards. Based on an application, the discounted reward may be omitted with *γ* = 0 to show the lack of dependency on future rewards, and this approach is generally called the myopic approach. As an example, Li [6] and Chen et al. [24] apply *Q*-learning in DCS (A1), and the *Q*-function in (1) is rewritten as follows:

(7) $$\begin{array}{c} Q_{t+1}^{i}\left(a_{t}^{i}\right)⟵\left(1-\alpha \right)Q_{t}^{i}\left(a_{t}^{i}\right)+\alpha \cdot r_{t+1}^{i}\left(a_{t}^{i}\right). \end{array}$$

### 3.5. *Q*-Function

The traditional *Q*-function (see (1)) has been widely applied to update *Q*-value in CR networks. The traditional *Q*-function can be enhanced in the context of CR as described next.

Lundén et al. [20] apply a linear function approximation-based approach to reduce the dimensionality of the large state-action spaces (or reduce the number of state-action pairs) in a collaborative channel sensing (A2) scheme. A linear function *f*(*s*_*t*_^*i*^, *a*_*t*_^*i*^) provides a matching value *θ*_*t*_(*s*_*t*_^*i*^, *a*_*t*_^*i*^) for a state-action pair. The matching value *θ*_*t*_(*s*_*t*_^*i*^, *a*_*t*_^*i*^), which shows the appropriateness of a state-action pair, is subsequently applied in *Q*-value computation. The linear function *f*(*s*_*t*_^*i*^, *a*_*t*_^*i*^) is normally fixed (or hard-coded), and various kinds of linear functions are possible to indicate the appropriateness of a state-action pair based on prior knowledge. For instance, *f*(*s*_*t*_^*i*^, *a*_*t*_^*i*^) yields a value that represents the level of desirability of a certain number of SU agents sensing a particular channel [20]. Higher *f*(*s*_*t*_^*i*^, *a*_*t*_^*i*^) value indicates that the number of SU agents sensing a particular channel is closer to a desirable number. Using a fixed linear function*f*(*s*_*t*_^*i*^, *a*_*t*_^*i*^), the learning problem is transformed into learning the matching value *θ*_*t*_(*s*_*t*_^*i*^, *a*_*t*_^*i*^) as follows:

(8) $$\begin{array}{c} Q_{t}^{i}\left(s_{t}^{i},a_{t}^{i}\right)=\theta_{t}\left(s_{t}^{i},a_{t}^{i}\right)\cdot f\left(s_{t}^{i},a_{t}^{i}\right). \end{array}$$

The parameter *θ*_*t*_(*s*_*t*_^*i*^, *a*_*t*_^*i*^)  is updated as follows:

(9) θt+1(sti,ati)=θt(sti,ati)+α[rt+1i(sti)+γ·Qti(st+1i,at+1i)−Qti(sti,ati)]·f(sti,ati).

### 3.6. Exploration and Exploitation

Traditionally, there are two popular approaches to achieve a balanced trade-off between exploration and exploitation, namely, softmax and *ε*-greedy [5]. For instance, Yau et al. [8] use the *ε*-greedy approach in which an agent explores with a small probability *ε* (i.e., *ε* = 0.1) and exploits with probability 1 − *ε*. Essentially, these approaches aim to control the frequency of exploration so that the best-known action is taken at most of the times. The traditional exploration and exploitation approach can be enhanced in the context of CR as described next.

In [3, 25], using the softmax approach, an agent selects actions based on a Boltzman distribution; specifically, the probability of selecting an action *a*_*t*_ in state *s*_*t*_ is as follows:

(10) $$\begin{array}{c} P\left(s_{t}^{i},a_{t}^{i}\right)=\frac{e^{Q_{t}^{i}({s_{t}^{i},a}_{t}^{i})/\tau_{t}}}{\sum_{j=1}^{K}e^{Q_{t}^{i}({s_{t}^{i},a}_{j}^{i})/\tau_{t}}}, \end{array}$$

where *τ*_*t*_ is a time-varying parameter called temperature. Higher temperature value indicates more exploration, while smaller temperature value indicates more exploitation. Denote the time duration during which exploration actions are being chosen by *T*_*e*_; the temperature *τ*_*t*_ is decreased as time goes by so that the agent performs more exploitation as follows:

(11) $$\begin{array}{c} \tau_{t}=-\frac{\left(\tau_{0}-\tau_{e}\right)\cdot t}{T_{e}}+\tau_{0}, \end{array}$$

where *τ*_0_ and *τ*_*e*_ are initial and final values of temperature, respectively. Note that, due to the dynamicity of the operating environment, exploration is necessary at all times, and so *τ*_*t*_ ≥ *τ*_0_.

In [21], using the *ε*-greedy approach, an agent uses a simple approach to decrease exploration probability as time goes by as follows:

(12) $$\begin{array}{c} \varepsilon_{t+1}=\max\left\{\delta \cdot \varepsilon_{t},\varepsilon_{\min}\right\}, \end{array}$$

where 0 ≤ *δ* ≤ 1 is a discount factor and *ε*_min⁡_ is the minimum exploration probability.

### 3.7. Other Features and Enhancements

This section presents other features and enhancements on the traditional RL approach found in various schemes for CR networks, including updates of learning rate, rules, and cooperative learning.

#### 3.7.1. Updates of Learning Rate

Traditionally, the learning rate *α* is a constant value [16]. The learning rate *α* may be adjusted as time goes by because higher value of *α* may compromise the RL algorithm's accuracy to converge to a correct action in a finite number of steps [26]. In [27], the learning rate reduces as time goes by using *α*(*t*) = *α*(*t* − 1) − Δ, where Δ is a small value to provide smooth transition between steps. In [14], the learning rate is updated using *α*(*t*) = Δ · *α*(*t* − 1).

#### 3.7.2. Rules

Rules determine a feasible set of actions for each state. The traditional RL algorithm does not apply rules although it is an important component in CR networks. For instance, in order to minimize interference with PUs, the SUs must comply with the timing requirements set by the PUs, such as the time interval that a SU must vacate its operating channel after any detection of PU activities.

As an example, Zheng and Li [15] propose an energy efficiency enhancement scheme in which there are four kinds of actions, namely, transmit, idle, sleep, and sense channel. Rules are applied so that the feasible set of actions is comprised of idle and sleep whenever the state indicates that there is no packet in the buffer. As another example, Peng et al. [4] propose a routing scheme, specifically, a next hop selection scheme in which the action represents the selection of a next hop out of a set of SU next hops. Rules are applied so that the feasible set of actions is limited to SU next hops with a certain level of SNR, as well as with shorter distance between next hop and the hop after next. The purposes of the rules are to reduce transmission delays and to ensure high-quality reception. Further description about [4, 15] is found in Table 1.

#### 3.7.3. Cooperative Learning

Cooperative learning enables neighbor agents to share information among themselves in order to expedite the learning process. The exchanged information can be applied in the computation of *Q*-function. The traditional RL algorithm does not apply cooperative learning, although it has been investigated in multiagent reinforcement learning (MARL) [28].

Felice et al. [11] propose a cooperative learning approach to reduce exploration. The *Q*-value is exchanged among the SU agents, and it is used in the *Q*-function computation to update *Q*-value. Each SU agent *i* keeps track of its own *Q*-value *Q*_*t*_^*i*^(*s*_*t*_^*i*^), and it is updated using the similar way to [6] (see Section 3.4). At any time instant, each agent *i* receives *Q*-value from its neighbor agent *j* ∈ *J* = {1,2,…,*N*_nbr,*i*_}. The agent keeps a vector of *Q*-value **Q**_**t**_^**i**^(**s**_**t**_^**i**^) with *s*_*t*_^*i*^ ∈ *S*. For the case *s*_*t*_^*j*^ = *s*_*t*_^*i*^, the *Q*-value *Q*_*t*_^*i*^(*s*_*t*_^*i*^) is updated as follows:

(13) $$\begin{array}{c} Q_{t}^{i}\left(s_{t}^{i}\right)=Q_{t}^{i}\left(s_{t}^{i}\right)+w\left(s_{t}^{i},j\right)\cdot \left(Q_{t}^{j}\left(s_{t}^{i}\right)-Q_{t}^{i}\left(s_{t}^{i}\right)\right), \end{array}$$

where *w*(*s*_*t*_^*i*^, *j*)  defines the weight assigned to cooperation with neighbor agent *j*. Similar approach has been applied in [25], and the *Q*-value *Q*_*t*_^*i*^(*s*_*t*_^*i*^) is updated based on the weight *w*(*s*_*t*_^*i*^, *j*) as follows:

(14) $$\begin{array}{c} Q_{t}^{i}\left(s_{t}^{i}\right)=\left(1-w\left(s_{t}^{i},j\right)\right)\cdot Q_{t}^{i}\left(s_{t}^{i}\right)+w\left(s_{t}^{i},j\right)\cdot Q_{t}^{j}\left(s_{t}^{i}\right). \end{array}$$

In [11], the weight *w*(*s*_*t*_^*i*^, *j*) depends on how much a neighbor agent *j* can contribute to the accurate estimation of value function *V*_*t*_^*i*^(*s*_*t*_^*i*^), such as the physical distance between agent *i* and *j*. In [25], the weight *w*(*s*_*t*_^*i*^, *j*) depends on the accuracy of the exchanged *Q*-value *Q*_*t*_^*i*^(*s*_*t*_^*i*^) (or expert value *E*_*t*_^*i*^(*s*_*t*_^*i*^) as described next) and the physical distance between agent *i* and *j*.

In [25], an agent exchanges its *Q*-value with its neighboring agents only if the expert value *E*_*t*_^*i*^(*s*_*t*_^*i*^) for *Q*-value *Q*_*t*_^*i*^(*s*_*t*_^*i*^) is greater than a particular threshold. The expert value *E*_*t*_^*i*^(*s*_*t*_^*i*^) indicates the accuracy of the *Q*-value *Q*_*t*_^*i*^(*s*_*t*_^*i*^). For instance, in [25], the *Q*-value *Q*_*t*_^*i*^(*s*_*t*_^*i*^) indicates the availability of white spaces in channel *s*_*t*_^*i*^, and so greater deviation in the signal strengths reduces the expert value *E*_*t*_^*i*^(*s*_*t*_^*i*^). By reducing the exchanges of *Q*-value with low accuracy, this approach reduces control overhead, and hence it reduces interference to PUs.

Application of cooperative learning in the CR context has been very limited. More description on cooperative learning is found in Section 4.8. Further research could be pursued to investigate how to improve network performance using this approach in existing and new schemes.

## 4. Reinforcement Learning in the Context of Cognitive Radio Networks: Models and Algorithms

Direct application of the traditional RL approach (see Section 2.1) has been shown to provide performance enhancement in CR networks. Reddy [29] presents a preliminary investigation in the application of RL to detect PU signals in channel sensing (A2). Table 1 presents a summary of the schemes that apply the traditional RL approach. For each scheme, we present the purpose(s) of the CR scheme, followed by its associated RL model.

Most importantly, this section presents a number of new additions to the RL algorithms, which have been applied to various schemes in CR networks. A summary of the new algorithms, their purposes, and references, is shown in Table 2. Each new algorithm has been designed to suit and to achieve the objectives of the respective schemes. For instance, the collaborative model (see Table 2) aims to achieve an optimal global reward in the presence of multiple agents, while the traditional RL approach achieves an optimal local reward in the presence of a single agent only. The following subsections (i.e., Sections 4.1–4.9) provide further details to each new algorithm, including the purpose(s) of the CR scheme(s), followed by its associated RL model (i.e., state, action, and reward representations) which characterize the purposes, and finally the enhanced algorithm which aims to achieve the purpose. Hence, these subsections serve as a foundation for further research in this area, particularly, the application of existing RL models and algorithms found in current schemes to either apply them in new schemes or extend the RL models in existing schemes to further enhance network performance.

### 4.1. Model 1: Model with *γ* = 0 in *Q*-Function

This is a myopic RL-based approach (see Section 3.4) that uses *γ* = 0 so that there is lack of dependency on future rewards, and it has been applied in [10, 17, 18]. Li et al. [10] propose a joint DCS (A1) and channel sensing (A2) scheme, and it has been shown to increase throughput, as well as to decrease the number of sensing channels (see performance metric (P4) in Section 5) and packet retransmission rate. The purposes of this scheme are to select operating channels with successful transmission rate greater than a certain threshold into a sensing channel set and subsequently to select a single operating channel for data transmission.

Table 3 shows the RL model for the scheme. The action *a*_*t*_^*i*^ ∈ *A*_*p*_ is to select whether to remain at the current operating channel or to switch to another operating channel with higher successful transmission rate. A preferred channel set *A*_*p*_ is composed of actions *a*_*t*_^*i*^ with *Q*-value *Q*_*t*_^*i*^(*a*_*t*_^*i*^) greater than a fixed threshold *Q*_th_ (e.g., *Q*_th_ = 5 in [10]). Since the state and action are similar in this model, the state representation is not shown in Table 3, and we represent *r*_*t*+1_^*i*^(*a*_*t*_^*i*^) = *r*_*t*+1_^*i*^(*s*_*t*+1_^*i*^). Note that *a*_*t*+1_^*i*^ = *a*_*t*_^*i*^ if there is no channel switch. The reward *r*_*t*+1_^*i*^(*a*_*t*_^*i*^) represents different kinds of events, specifically, *r*_*t*+1_^*i*^(*a*_*t*_^*i*^) = 1 in case of successful transmission, and *r*_*t*+1_^*i*^(*a*_*t*_^*i*^) = −1 in case of unsuccessful transmission or channel *a*_*t*+1_^*i*^ is sensed busy. The RL model is embedded in a centralized entity such as a base station.

Algorithm 1 presents the RL algorithm for the scheme. The action *a*_*t*_^*i*^ ∈ *A*_*p*_ is chosen from a preferred channel set. The update of the *Q*-value *Q*_*t*+1_^*i*^(*a*_*t*_^*i*^) is self-explanatory. Similar approach has been applied in DCS (A1) [30, 31].

Li et al. [18] propose a MAC protocol, which includes both DCS (A1) and a retransmission policy (A6), to minimize channel contention. The DCS scheme enables the SU agents to minimize their possibilities of operating in the same channel. This scheme uses the RL algorithm in Algorithm 1, and the reward representation is extended to more than a single performance enhancement. Specifically, the reward *r*_*t*+1_^*i*^(*a*_*t*_^*i*^) represents the successful transmission rate and transmission delay. Higher reward indicates higher successful transmission rate and lower transmission delay, and vice versa. To accommodate both transmission rate and transmission delay in *Q*-function, the reward representation becomes *r*_*t*+1_^*i*^(*a*_*t*_^*i*^) = *r*_*t*+1_^*i*,′^(*a*_*t*_^*i*^) + *r*_*t*+1_^*i*,′′^(*a*_*t*_^*i*^), and so the *Q*-function becomes *Q*_*t*+1_^*i*^(*a*_*t*_^*i*^) = *Q*_*t*_^*i*^(*a*_*t*_^*i*^) + *r*_*t*+1_^*i*,′^(*a*_*t*_^*i*^) + *r*_*t*+1_^*i*,′′^(*a*_*t*_^*i*^). The retransmission policy determines the probability a SU agent transmits at time *t*, and so *Q*_*t*+1_^*i*^(*a*_*t*_^*i*^) indicates the probability a SU agent transmits at time *t*. The reward *r*_*t*+1_^*i*,′^(*a*_*t*_^*i*^) = 1, 0, and −1 if the transmission delay at time *t* is smaller than, equal to, and greater than the average transmission delay, respectively. The reward *r*_*t*+1_^*i*,′′^(*a*_*t*_^*i*^) represents different kinds of events; specifically, *r*_*t*+1_^*i*,′′^(*a*_*t*_^*i*^) = 2, 0, and −2 in case of successful transmission, idle transmission, and unsuccessful transmission, respectively; note that idle indicates that channel *a*_*t*_^*i*^ is sensed busy, and so there is no transmission.

Li et al. [17] propose a MAC protocol (A6) to reduce the probability of packet collision among PUs and SUs, and it has been shown to increase throughput and to decrease packet loss rate. Since both successful transmission rate and the presence of idle channels are important factors, it keeps track of the *Q*-functions for channel sensing *Q*_*t*_^*i*^(*a*_se_^*i*^) and transmission *Q*_*t*_^*i*^(*a*_tx_^*i*^) using RL algorithm in Algorithm 1, respectively. Hence, similar to Algorithm 2 in Section 4.2, there is a set of two *Q*-functions. The action *a*_*t*_^*i*^ is to select whether to remain at the current operating channel or to switch to another operating channel. The sensing reward *r*_*t*+1_^*i*^(*a*_se_^*i*^) = 1 and −1 if the channel is sensed idle and busy, respectively. The transmission reward *r*_*t*+1_^*i*^(*a*_tx_^*i*^) = 1 and −1 if the transmission is successful and unsuccessful, respectively. Action selection is based on the maximum average *Q*-value; specifically, *Q*_*t*_^*i*^(*a*_*t*_^*i*^) = [*Q*_*t*_^*i*^(*a*_se_^*i*^) + *Q*_*t*_^*i*^(*a*_tx_^*i*^)]/2.

### 4.2. Model 2: Model with a Set of *Q*-Functions

A set of distinctive *Q*-functions can be applied to keep track of the *Q*-value of different actions, and it has been applied in [11, 21]. Di Felice et al. [11] propose a joint DCS (A1) and channel sensing (A2) scheme, and it has been shown to increase goodput and packet delivery rate, as well as to decrease end-to-end delay and interference level to PUs. The purposes of this scheme are threefold:

- firstly, it selects an operating channel that has the lowest channel utilization level by PUs;
- secondly, it achieves a balanced trade-off between the time durations for data transmission and channel sensing;
- thirdly, it reduces the exploration probability using a knowledge sharing mechanism.

Table 4 shows the RL model for the scheme. The state *s*_*t*_^*i*^ ∈ *S* represents a channel for data transmission. The actions *a*_*t*_^*i*^ ∈ *A* are to sense channel, to transmit data, or to switch its operating channel. The reward *r*_*t*+1_^*i*^(*s*_*t*+1_^*i*^) represents the difference between two types of delays, namely, the maximum allowable single-hop transmission delay and a successful single-hop transmission delay. A single-hop transmission delay covers four kinds of delays including backoff, packet transmission, packet retransmission, and propagation delays. Higher reward level indicates shorter delay incurred by a successful single-hop transmission. The RL model is embedded in a centralized entity such as a base station.

Algorithm 2 presents the RL algorithm for the scheme. Denote learning rate by 0 ≤ *α* ≤ 1, eligible trace by *e* ^*i*^(*s*_*t*_^*i*^), and the amount of time during which the SU agent is involved in successful transmissions or was idle (i.e., no packets to transmit) by *T*_*u*_^*i*^, as well as the temporal differences by (*T*_se_^*i*^ − *Q*_*t*_^*i*^(*s*_*t*_^*i*^, *a*_se_^*i*^)) and (*T*_*u*_^*i*^ − *Q*_*t*_^*i*^(*s*_*t*_^*i*^, *a*_tx_^*i*^)). A single type of *Q*-function is chosen to update the *Q*-value *Q*_*t*_^*i*^(*s*_*t*_^*i*^, *a*_*t*_^*i*^) based on the current action *a*_*t*_^*i*^ ∈ *A* = {*a*_se_^*i*^, *a*_tx_^*i*^, *a*_sw_^*i*^} being taken. The temporal difference indicates the difference between the actual outcome and the estimated *Q*-value.

In step (b), the eligible trace *e* ^*i*^(*s*_*t*_^*i*^) represents the temporal validity of state *s*_*t*_^*i*^. Specifically, in [11], eligible trace *e* ^*i*^(*s*_*t*_^*i*^) represents the existence of PU activities in channel *s*_*t*_^*i*^, and so it is only updated when channel sensing operation *a*_se_^*i*^  is taken. Higher eligible trace *e* ^*i*^(*s*_*t*_^*i*^) indicates greater presence of PU activities, and vice versa. Hence, the term *e* ^*i*^(*s*_*t*_^*i*^) is in the update of *Q*-value *Q*_*t*+1_^*i*^(*s*_*t*_^*i*^, *a*_se_^*i*^), and (1 − *e* ^*i*^(*s*_*t*_^*i*^))is in the update of *Q*-value *Q*_*t*+1_^*i*^(*s*_*t*_^*i*^, *a*_tx_^*i*^) in Algorithm 2. Therefore, higher eligible trace *e* ^*i*^(*s*_*t*_^*i*^) results in higher value of *Q*_*t*+1_^*i*^(*s*_*t*_^*i*^, *a*_se_^*i*^) and lower value of *Q*_*t*+1_^*i*^(*s*_*t*_^*i*^, *a*_tx_^*i*^), and this indicates more channel sensing tasks and lesser data transmission in channels with greater presence of PU activities. The action *a*_sw_^*i*^ switches channel from state *s*_*t*_^*i*^ to state *s*_*t*+1_^*i*^. The *ε*-greedy approach is applied to choose the next channel *s*_*t*+1_^*i*^. In [21], eligible trace *e* ^*i*^(*s*_*t*_^*i*^), which represents the temporal validity or freshness of the sensing outcome, is only updated when the channel sensing operation *a*_se_^*i*^  is taken as shown in Algorithm 2. The eligible trace *e* ^*i*^(*s*_*t*_^*i*^) is discounted whenever *a*_se_^*i*^ is not chosen as follows:

(15) $$\begin{array}{c} e^{i}\left(s_{t+1}^{i}\right)=\{\begin{array}{cc} 1, & \text{if}\;a_{t}^{i}=a_{\text{se}}^{i}\\ \delta \cdot e^{i}\left(s_{t}^{i}\right), & \text{otherwise}, \end{array} \end{array}$$

where 0 ≤ *δ* ≤ 1 is a discount factor for the eligible trace. Equation (15) shows that the eligible trace of each state *s*_*t*_^*i*^ is set to the maximum value of 1 whenever action *a*_se_^*i*^ is taken; otherwise, it is decreased with a factor of *δ*.

In step (c), the *Q*_*t*_^*i*^(*s*_*t*_^*i*^, *a*_sw_^*i*^) value keeps track of the channel that provides the best-known lowest estimated average transmission delay. In other words, the channel must provide the maximum amount of reward that can be achieved considering the cost of a channel switch *θ*. Hence, *Q*_*t*_^*i*^(*s*_*t*_^*i*^, *a*_sw_^*i*^) can keep track of a channel *s*_*t*+1_^*i*^ that provides the best-known state value *V*_*t*_^*i*^(*s*_*t*+1_^*i*^) the SU agent receives compared to the average state value *V*_*t*_^*i*^(*s*) by switching its current operating channel *s*_*t*_^*i*^ to the operating channel *s*_*t*+1_^*i*^. Note that the state value *V*_*t*_^*i*^(*s*_*t*_^*i*^) is exchanged among the SU agents to reduce exploration through cooperative learning (see Section 3.7.3).

In step (d), the policy *π* ^*i*^(*s*_*t*+1_^*i*^, *a*_*t*+1_^*i*^) is applied at the next time instant. The policy provides probability distributions over the three possible types of actions *A* = {*a*_se_^*i*^, *a*_tx_^*i*^, *a*_sw_^*i*^} using a modified Boltzmann distribution (see Section 3.6). Next, the policy is applied to select the next action *a*_*t*+1_^*i*^ in step (a).

### 4.3. Model 3: Dual *Q*-Function Model

The dual *Q*-function model has been applied to expedite the learning process [32]. The traditional *Q*-function (see (1)) updates a single *Q*-value at a time, whereas the dual *Q*-function updates two *Q*-values simultaneously. For instance, in [33], the traditional *Q*-function updates the *Q*-value for the next state only (e.g., SU destination node), whereas the dual *Q*-function updates the *Q*-value for the next and previous states (e.g., SU source and destination nodes, respectively). The dual *Q*-function model updates a SU agent's *Q*-value in both directions (i.e., towards the source and destination nodes) and speeds up the learning process in order to make more accurate decisions on action selection; however, at the expense of higher network overhead incurred by more *Q*-value exchanges among the SU neighbor nodes.

Xia et al. [33] propose a routing (A7) scheme, and it has been shown to reduce SU end-to-end delay. Generally speaking, the availability of channels in CR networks is dynamic, and it is dependent on the channel utilization level by PUs. The purpose of this scheme is to enable a SU node to select a next-hop SU node with higher number of available channels. The higher number of available channels reduces the time incurred in seeking for an available common channel for data transmission among a SU node pair, and hence it reduces the MAC layer delay.

Table 5 shows the RL model for the scheme. The state *s*_*t*_^*i*^ ∈ *S* represents a SU destination node *n*. The action *a*_*t*_^*i*^ ∈ *A* represents the selection of a next-hop SU neighbor node *j*. The reward *r*_*t*+1_^*i*^(*s*_*t*+1_^*i*^, *a*_*t*+1_^*i*^) represents the number of available common channels among nodes *i* and *a*_*t*_^*i*^ = *j*. The RL model is embedded in each SU agent.

This scheme applies the traditional *Q*-function (see (1)) with *γ* = 1. Hence, the *Q*-function is rewritten as follows:

(16) Qt+1i(sti,j)⟵(1−α)Qti(sti,j)  +α[rt+1i(st+1i,j)+max⁡k ∈atj⁡Qtj(st+1i,k)],

where *k* ∈ *a*_*t*_^*j*^ is an upstream node of SU neighbor node *j*, so node *j* must estimate and send information on max⁡_*k *∈*a*_*t*_^*j*^_⁡*Q*_*t*_^*j*^(*s*_*t*+1_^*i*^, *k*) to SU node *i*.

The dual *Q*-function model in this scheme is applied to update the *Q*-value for the SU source and destination nodes. While the traditional *Q*-function enables the SU intermediate node to update the *Q*-value for the SU destination node only (or next state), which is called forward exploration, the dual *Q*-function model enables an intermediate SU node to achieve backward exploration as well by updating the *Q*-value for the SU source node (or previous state). Forward exploration is achieved by updating the *Q*-value at SU node *i* for the SU destination node whenever it receives an estimate max⁡_*k *∈*a*_*t*_^*j*^_⁡*Q*_*t*_^*j*^(*s*_*t*+1_^*i*^, *k*)  from SU node *j*, while backward exploration is achieved by updating the *Q*-value at SU node *j* for the SU source node whenever it receives a data packet from node *i*. Note that, in the backward exploration case, node *i*'s packets are piggybacked with its *Q*-value so that node *j* is able to update *Q*-value for the respective SU source node. Although the dual *Q*-function approach increases the network overhead, it expedites the learning process since SU nodes along a route update *Q*-value of the route in both directions.

### 4.4. Model 4: Partial Observable Model

The partial observable model has been applied in a dynamic and uncertain operating environment. The uniqueness of the partial observable model is that the SU agents are uncertain about their respective states, and so each of them computes belief state *b*(*s*_*t*_^*i*^), which is the probability that the environment is operating in state *s*_*t*_^*i*^.

Bkassiny et al. [34] propose a joint DCS (A1) and channel sensing (A2) scheme, and it has been shown to improve the overall spectrum utilization. The purpose of this scheme is to enable the SU agents to select their respective operating channels for sensing and data transmission in which the collisions among the SUs and PUs must be minimized.

Table 6 shows the RL model for the scheme. The state **s**_**t**_^**i**^ ∈ *S*_1_ × *S*_2_ × ⋯×*S*_*K*_ represents the availability of a set of channels for data transmission. The action *a*_*t*_^*i*^ ∈ *A* represents a single channel out of *K* channels available for data transmission. The reward represents fixed positive (negative) values to be rewarded (punished) for successful (unsuccessful) transmissions. The RL model is embedded in each SU agent so that it can make decision in a distributed manner.

Algorithm 3 presents the RL algorithm for the scheme. The action *a*_*t*_^*i*^ ∈ *A* is chosen from a preferred channel set. The chosen action has the maximum belief-state *Q*-value, which is calculated using belief vector **b**(**s**_**t**_^**i**^) = (*b*(*s*_1,*t*_^*i*^), *b*(*s*_2,*t*_^*i*^),…, *b*(*s*_*K*,*t*_^*i*^)) as weighting factor. The belief vector **b**(**s**_**t**_^**i**^) is the probability of a possible set of state **s**_**t**_^**i**^ = (*s*_1,*t*_^*i*^, *s*_2,*t*_^*i*^,…, *s*_*K*,*t*_^*i*^) being idle at time *t* + 1. Upon receiving reward *r*_*t*+1_^*i*^(**s**_**t**+1_^**i**^, *a*_*t*_^*i*^), the SU agent updates the entire set of belief vectors **b**(**s**_**t**_^**i**^) using Bayes' formula [34]. Next, the SU agent updates the *Q*-value *Q*_*t*+1_^*i*^(**s**_**t**_^**i**^, *a*_*t*_^*i*^). Note that max⁡_*a*∈*A*_ *Q*_*b*,*t*+1_^*i*^(**s**_**t**+1_^**i**^, *a*) = max⁡_*a*∈*A*_∑_*s*∈**s**_**t**_^**i**^_ *b*(*s*)*Q*_*t*_^*i*^(*s*, *a*).

It shall be noted that Bkassiny et al. [34] apply the belief vector **b**(**s**_**t**_^**i**^) as a weighting vector in its computation of *Q*-value *Q*_*t*+1_^*i*^(**s**_**t**_^**i**^, *a*_*t*_^*i*^), while most of the other approaches, such as [20], use belief vector **b**(**s**_**t**_^**i**^) as the actual state, specifically, *Q*_*t*+1_^*i*^(**b**(**s**_**t**_^**i**^), *a*_*t*_^*i*^). This approach has been shown to achieve a near-optimal solution with a very low complexity in [35].

### 4.5. Model 5: Actor-Critic Model

Traditionally, the delayed reward has been applied directly to update the *Q*-value. The actor-critic model adjusts the delayed reward value using reward corrections, and this approach has been shown to expedite the learning process. In this model, an actor selects actions using suitability value, while a critic keeps track of temporal difference, which takes into account reward corrections in delayed rewards.

Vucevic et al. [13] propose a collaborative channel sensing (A2) scheme, and it has been shown to minimize error detection probability in the presence of inaccurate sensing outcomes. The purpose of this scheme is that it selects neighboring SU agents that provide accurate channel sensing outcomes for security enhancement purpose (A3).  Table 7 shows the RL model for the scheme. The state is not represented. An action *a*_*t*_^*i*^ ∈ *A* represents a neighboring SU chosen by SU agent *i* for channel sensing purpose. The reward *r*_*t*+1_^*i*^(*a*_*t*_^*i*^) represents fixed positive (negative) values to be rewarded (punished) for correct (incorrect) sensing outcomes compared to the final decision, which is the fusion of the sensing outcomes. The RL model is embedded in each SU agent.

The critic keeps track of *c*_*t*+1_^*i*^(*a*_*t*_^*i*^) = *c*_*t*_^*i*^(*a*_*t*_^*i*^) + *β* · Δ*c*_*t*_^*i*^(*a*_*t*_^*i*^), where Δ*c*_*t*_^*i*^(*a*_*t*_^*i*^) is the temporal difference and *β* is a constant (e.g., *β* = 0.01). In [13], Δ*c*_*t*_^*i*^(*a*_*t*_^*i*^) depends on the difference between the delayed reward *r*_*t*+1_^*i*^(*a*_*t*_^*i*^) and the long-term delayed reward $\overset{-}{r_{t+1}^{i}}\left(a_{t}^{i}\right)=\alpha \cdot r_{t+1}^{i}\left(a_{t}^{i}\right)+\left(1-\alpha \right)\cdot \overset{-}{r_{t}^{i}}\left(a_{t}^{i}\right)$, the number of incorrect sensing outcomes, and the suitability value *π*_*t*_^*i*^(*a*_*t*_^*i*^). Next, the actor selects actions using *c*_*t*+1_^*i*^(*a*_*t*_^*i*^) given by the critic. The probability of selecting action *a*_*t*_^*i*^ is based on the suitability value of action *i*; *π*_*t*_^*i*^(*a*_*t*_^*i*^) = *e* ^*c*_*t*+1_^*i*^(*a*_*t*_^*i*^)^/∑_*a*∈*N*_nbr,*i*__ *e* ^*c*_*t*+1_^*i*^(*a*)^.

### 4.6. Model 6: Auction Model

The auction model has been applied in centralized CR networks. In the auction model, a centralized entity, such as a base station, conducts auctions and allows SU hosts to place bids so that the winning SU hosts receive rewards. The centralized entity may perform simple tasks, such as allocating white spaces to SU hosts with winning bids [16], or it may learn using RL to maximize its utility [36]. The RL model may be embedded in each SU host in a centralized network [16, 36–38], or in the centralized entity only [36].

Chen and Qiu [16] propose a channel auction scheme (A5), and it has been shown to allocate white spaces among SU hosts (or agents) efficiently and fairly. The purpose of this scheme is to enable the SU agents to select the amount of bids during an auction, which is conducted by centralized entity, for white spaces. The SU agents place the right amount of bids in order to secure white spaces for data transmission, while saving their credits, respectively. The RL model is embedded in each SU host.

Table 8 shows the RL model for the scheme. The state *s*_*t*_^*i*^ ∈ *S* indicates a SU agent's information, specifically, the amount of data for transmission in its buffer and the amount of credits (or “wealth”) it owns. The action *a*_*t*_^*i*^ ∈ *A* is the amount of a bid for white spaces. The reward *r*_*t*+1_^*i*^(*s*_*t*+1_^*i*^) indicates the amount of data sent. This scheme applies the traditional *Q*-learning approach (see (1)), to update *Q*-values.

Jayaweera et al. [36] propose another channel auction scheme (A5) that allocates white spaces among SUs, and it has been shown to increase transmission rates of the SUs and to reduce energy consumption of the PUs. In [36], the PUs adjust the amount of white spaces and allocate them to the SUs with winning bids. The winning SUs transmit their packets, as well as relaying PUs' packets using the white spaces so that the PUs can reduce its energy consumption. In other words, the SUs use their power as currency to buy the bandwidth. Two different kinds of RL models are embedded in PUs and SUs, respectively, so that the PUs can learn to adjust the amount of white spaces to be allocated to the SUs, and the SUs can learn to select the amount of bids during an auction for white spaces.

The state is not represented, and we show the action and reward representations of the scheme.  Table 9 shows the reward representation of the RL model. The reward *r*_*t*+1_^*i*^(*a*_*k*,*t*_^*i*^) indicates a constant positive reward in case of successful bid and a constant negative reward in case of unsuccessful bid. The reward representation is embedded in both PUs and SUs. The actions for both PUs and SUs are different. Each SU *i* selects the amount of bid *a*_*k*,*t*_^*i*^ ∈ *A* during an auction for white spaces in channel *k*, while each PU adjusts the amount of white spaces *a*_*k*,*t*_^*i*^ ∈ *A* to be offered for auction in its own channel *k*. Higher amount of white spaces encourages the SUs to participate in auctions.

This scheme applies *Q*-function *Q*_*k*,*t*+1_^*i*^(*a*_*k*,*t*_^*i*^) = *Q*_*k*,*t*_^*i*^(*a*_*k*,*t*_^*i*^) + *r*_*t*+1_^*i*^(*a*_*k*,*t*_^*i*^) with *γ* = 0 (see Section 4.1) at both PUs and SUs. The SUs' *Q*-function indicates the appropriate amount of bids for white spaces, while the PUs' *Q*-function indicates the appropriate amount of white spaces to be offered for auction.

Fu and Van der Schaar [37] propose a channel auction scheme (A5) that improves the bidding policy of SUs, and it has been shown to reduce SUs' packet loss rate. The purpose of this scheme is to enable SU agents to learn and adapt the amount of bids during an auction for time-varying white spaces in dynamic wireless networks with environmental disturbance and SU-SU disturbance. Examples of* environmental disturbance* are dynamic level of channel utilization by PUs, channel condition (i.e., SNR), and SU traffic rate, while an example of SU-SU disturbance is the effect from other competing SUs, who are noncollaborative and autonomous in nature. Compared to traditional centralized auction schemes, SUs compute their bids based on their knowledge and observation of the operating environment with limited information received from other SUs and the centralized base station. Note that the joint bidding actions of SUs affect the allocation of white spaces and bidding policies of the other SUs, and so the proposed learning algorithm improves the bidding policy of SUs based on the observed white space allocations and rewards.

Table 10 shows the RL model for the scheme. The state *s*_*t*_^*i*^ ∈ *S* indicates SU agent's information, specifically, its buffer state, as well as the states of the available channels in terms of SNR. The action *a*_*t*_^*i*^ ∈ *A* is the amount of bids for white spaces. The reward *r*_*t*+1_^*i*^(**s**_*t*+1,_ **w**_*t*+1,_ **a**_*t*+1  _) represents the sum of the number of lost packets *g*_*t*+1_^*i*^ and the channel cost *c*_*t*+1_^*i*^ that SU *i* must pay for using the channel. Note that the channel cost *c*_*t*+1_^*i*^ represents network congestion, and hence higher cost *c*_*t*+1_^*i*^ indicates higher congestion level. The RL model is embedded in each SU host.

Algorithm 4 presents the RL algorithm for the scheme. In step (a), SU agent *i* observes its current state *s*_*t*_^*i*^ and available channels (or white spaces) **w**_*t*_ advertised by the centralized base station. In step (b), it decides and submits its bids to the base station, and the bids are estimated based on SU *i*'s state *s*_*t*_^*i*^ and other SUs' representative (or estimated) state ${\tilde{s}}_{t}^{-i}$. Note that, since SU *i* needs to know all the states and transition probabilities of other SUs, which may not be feasible, it estimates the representative state ${\tilde{s}}_{t}^{-i}$ based on its previous knowledge of channel allocation *z*_*t*_^*i*^ and channel cost *c*_*t*+1_^*i*^ (or network congestion). In step (c), SU *i* receives its channel allocation decision *z*_*t*_^*i*^ and the required channel cost *c*_*t*_^*i*^ from the base station. In step (d), the representative state ${\tilde{s}}_{t}^{-i}$ and transition probabilities ${\tilde{p}}_{t}^{-i}$ of the other SUs are updated based on the newly received channel allocation decision *z*_*t*_^*i*^ and the required channel cost *c*_*t*_^*i*^ information. In step (e), SU *i* computes its estimated *Q*-value, which is inspired by the traditional *Q*-function approach, and this approach explicitly takes into account the effects of the bidding actions of the other SUs based on their estimated representative state ${\tilde{s}}_{t}^{-i}$ and transition probabilities ${\tilde{p}}_{t}^{-i}$. Note that **a**_*t*_ also denotes Markov-based policy profile that representsthe bidding policies of all the other SUs. In step (f), the *Q*-table is updated if there are changes in the SU states and channel availability.

Xiao et al. [38] propose a power control scheme (A8), and it has been shown to increase the transmission rates and payoffs of SUs. There are two main differences compared to the traditional auction schemes, which have been applied to centralized networks. Firstly, the interactions among all nodes, including PUs and SUs, are coordinated in a distributed manner. A SU source node transmits its packets to the SU destination node using either single-hop transmission or multihop relaying. In multihop relaying, a SU source node must pay the upstream node, which helps to relay the packets. Secondly, the PUs treat each SU equally, and so there is lack of competitiveness in auctions. Each SU may accumulate credits through relaying. Game theory is applied to model the network in which SUs pay credits to PUs for using licensed channels and to other SUs for relaying their packets. The purpose of this scheme is to enable a SU node to choose efficient actions in order to improve its payoff, as well as to collect credits through relaying, and to minimize the credits paid to PUs and other SU relays. A RL model is embedded in each SU.

The state is not represented, and we show the action and reward representations of the scheme.  Table 11 shows the RL model for the scheme. The action *a*_*t*_^*i*^ ∈ *A* represents transmission of SU *i*'s packets by either using single-hop transmission or multihop relaying. The reward *r*_*t*+1_^*i*^(*a*_*t*_^*i*^) indicates the revenue (or profit) received by SU node *i* for providing relaying services to other SUs, and so higher reward indicates higher transmission rate and increased transmission power of SU node *i*. Denote the payoff of SU *i* by *p*_*t*_^*i*^, as shown in (17). The payoff indicates the difference between SU *i*'s revenue and costs. There are two types of costs represented by *c*_*t*_^*i*,*j*^and *c*_*t*_^*i*,PU^. The *c*_*t*_^*i*,*j*^ represents the cost charged by the upstream SU node *j* for relaying SU node *i*'s packets, and the *c*_*t*_^*i*,PU^ represents the cost charged by all PUs for using the white spaces in licensed channels. The *c*_*t*_^*i*,PU^ increases with the SU *i*'s interference power in the respective channel. Consider

(17) $$\begin{array}{c} p_{t}^{i}=\overset{N}{\underset{j=1}{\sum }}\left(r_{t}^{i,j}+c_{t}^{i,j}{+\;c}_{t}^{i,\text{PU}}\right). \end{array}$$

This scheme applies *Q*-function *Q*_*t*+1_^*i*^(*a*_*t*_^*i*^) = *Q*_*t*_^*i*^(*a*_*t*_^*i*^) + *δ*(*p*_*t*_^*i*^ · *P*_*t*_^*i*^(*a*_*t*_^*i*^)), which indicates the average payoff, where *δ* is a constant step size and *P*_*t*_^*i*^(*a*_*t*_^*i*^) is the probability of SU *i* choosing action *a*_*t*_^*i*^, which is computed according to Boltzmann distribution (see Section 3.6).

### 4.7. Model 7: Internal Self-Learning Model

The internal self-learning model has been applied to expedite the learning process. The uniqueness of the internal self-learning model lies in the learning approach in which the learning mechanism continuously interacts with a simulated internal environment within the SU agent itself. The learning mechanism continuously exchanges its actions with rewards generated by the simulated internal environment so that the SU agent learns the optimal actions for various settings of the operating environment, and this helps *Q*-value and the optimal action to converge.

Bernardo et al. [27] propose a DCS (A1) scheme, and it has been shown to improve the overall spectrum utilization and throughput performances. Note that, unlike the previous schemes in which the RL models are embedded in the SU agents, the RL model is embedded in each PU base station (or agent) in this scheme, and it is applied to make medium-term decisions (i.e., from tens of seconds to tens of minutes). The purpose of this scheme is to enable a PU agent to select its operating channels for transmission in its own cell. In order to improve the overall spectrum utilization, the PU agent preserves its own QoS while generating white spaces and sells them off to SU agents.

Table 12 shows the RL model for the scheme. The action **a**_**t**_^**i**^ ∈ *A*_1_ × *A*_2_ × ⋯×*A*_*K*_ is a set of chosen available channels for the entire cell. The reward *r*_*t*+1_^*i*^(**a**_**t**_^**i**^) has a zero value if the estimated throughput of an action selection **a**_**t**_^**i**^ is less than a throughput threshold *T*_th_; otherwise, the reward is based on the spectrum efficiency $\hat{\eta }\left(a_{t}^{i}\right)$ and the amount of white spaces $\overset{-}{W}\left(a_{t}^{i}\right)$, which may be sold off to SU agents. Both *λ* and *μ* are constant weight factors.

**Figure 3:**
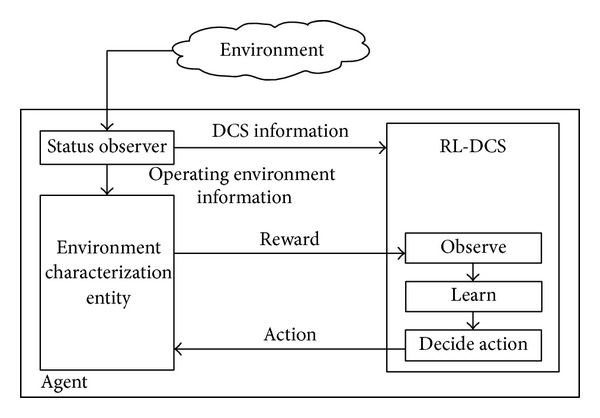
Internal self-learning model.

Figure 3 shows the internal self-learning model. The learning mechanism, namely, RL-DCS, continuously interacts with a simulated internal environment, namely, Environment Characterization Entity (ECE). Based on the information observed from the real operating environment (i.e., the number of PU hosts and the average throughput per PU host), which is provided by status observer, the ECE implements a model of the real operating environment (i.e., spectrum efficiency $\hat{\eta }\left(a_{t}^{i}\right)$ and the amount of white spaces $\overset{-}{W}\left(a_{t}^{i}\right)$) and computes reward *r*_*t*+1_^*i*^(**a**_**t**_^**i**^). Hence, the ECE evaluates the suitability of action **a**_**t**_^**i**^ in its simulated internal model of the operating environment. By exchanging action **a**_**t**_^**i**^ and reward *r*_*t*+1_^*i*^(**a**_**t**_^**i**^) between RL-DCS and ECE, the RL-DCS learns an optimal action **a**_**t**_^**i**^ at a faster rate compared to the conventional learning approach, and this process stops when the optimal action **a**_**t**_^**i**^ converges.

Algorithm 5 presents the RL algorithm for the scheme. The action **a**_**t**_^**i**^ = (*a*_1,*t*_^*i*^, *a*_2,*t*_^*i*^,…, *a*_*K*,*t*_^*i*^) ∈ *A*_1_ × *A*_2_ × ⋯×*A*_*K*_ is chosen using a Bernoulli random variable [27]. The PU agent receives reward *r*_*t*+1_^*i*^(**a**_**t**_^**i**^) computed by ECE and computes the average reward ${\overset{-}{r}}_{t}^{i}\left(a_{k,t}^{i}\right)$ for each subaction *a*_*k*,*t*_^*i*^ at time *t* using the exponential moving average [27]. Denote the probability of taking action *a*_*k*,*t*_^*i*^ by *P*_*t*_^*i*^(*a*_*k*,*t*_^*i*^) and the current overall unused spectrum, which is the ratio of the unused bandwidth to the total bandwidth of a cell, by *x*. Upon receiving reward *r*_*t*+1_^*i*^(**a**_**t**_^**i**^), the PU agent updates the *Q*-value *Q*_*t*+1_^*i*^(*a*_*k*,*t*_^*i*^) for each action *a*_*k*,*t*_^*i*^∈**a**_**t**_^**i**^. Finally, the probability of taking action *a*_*k*,*t*_^*i*^, specifically, *P*_*t*_^*i*^(*a*_*k*,*t*_^*i*^), is updated. Note that the exploration probability is *ε*.

### 4.8. Model 8: Collaborative Model

Collaborative model enables a SU agent to collaborate with its SU neighbor agents and subsequently make local decisions independently in distributed CR networks. It enables the agents to learn and achieve an optimal joint action. A joint action is defined as the actions taken by all the agents throughout the entire network. An optimal joint action is the actions taken by all the agents throughout the entire network that provides an ideal and optimal network-wide performance. Hence, the collaborative model reduces the selfishness of each agent through taking other agents' actions or strategies into account. The collaboration may take the form of exchanging local information, including knowledge (*Q*-value), observations, and decisions, among the SU agents.

Lundén et al. [20] propose a collaborative channel sensing (A2) scheme, and it has been shown to maximize the amount of white spaces found. The purposes of this scheme are twofold:

- firstly, it selects channels with more white spaces for channel sensing purpose;
- secondly, it selects channels so that the SU agents diversify their sensing channels. In other words, the SU agents perform channel sensing in various channels.

Table 13 shows the RL model for the scheme. The state **s**_**t**_^**i**^ ∈ *S*_1_ × *S*_2_ × ⋯×*S*_*K*_ represents the belief on the availability of a set of channels for data transmission. An action *a*_*t*_^*i*^ ∈ *A*, which is part of the joint action **a**_**t**_ representing all the actions taken by SU agent *i* and its SU neighbor agents, represents a single channel chosen by SU agent *i* for channel sensing purpose. The reward *r*_*t*+1_^*i*^(**s**_**t**+1_^**i**^, *a*_*t*_^*i*^) represents the number of channels identified as being idle (or free) at time *t* + 1 by SU agent *i*. The RL model is embedded in each SU agent.

Algorithm 6 presents the RL algorithm for the scheme, and it is comprised of two rounds of collaboration message exchanges. After taking action *a*_*t*_^*i*^ ∈ *A*, the SU agent *i* exchange collaboration messages *D*_*t*,1_^*i*^ = (*a*_*t*_^*i*^, *β*_*t*_^*i*^) with its SU neighbor agents. The *D*_*t*,1_^*i*^ is comprised of two-tuple information, namely, SU agent *i*'s action *a*_*t*_^*i*^ and SU agent *i*'s sensing outcomes *β*_*t*_^*i*^. SU agent *i* determines the delayed reward based on *D*_*t*,1_^*i*^. Next, the SU agent *i* exchanges collaboration messages *D*_*t*,2_^*i*^ = *a*_*t*+1_^*i*^ with its SU neighbor agents. During the second round of collaboration message exchange, a SU agent *i* chooses its action *a*_*t*+1_^*i*^ for the next time instance upon receiving *D*_*t*,2_^*j*^ from SU neighbor agent *j*. Note that the SU agent transmission order affects the action selection. This is because a SU agent may receive and use information obtained from its preceding agents, and so it can make decisions using more updated information in the second round. Since one of the main purposes is to enable the SU agents to diversify their sensing channels, the SU agents choose action *a*_*t*+1_^*i*^ from a preferred channel set. The preferred channel set is comprised of sensing channels which are yet to be chosen by the preceding SU agents. The SU agent chooses channels with the maximum *Q*-value from the preferred channel set. Finally, the SU agent updates *Q*-value *Q*_*t*+1_^*i*^(**s**_**t**_^**i**^, *a*_*t*_^*i*^) and *θ*_*t*+1_(*s*_*t*_^*i*^, *a*_*t*_^*i*^) (see Section 3.5).

Liu et al. [39] propose a collaborative DCS (A1) scheme that applies a collaborative model, and it has been shown to achieve a near-optimal throughput performance. The purpose of this scheme is to enable each SU link to maximize its individual delayed rewards, specifically, the SNR level. Note that this collaboration approach assumes that an agent has full observation of the actions and policies adopted by all the other SU links at any time instance. Hence, (1) is rewritten as follows:

(18) Qt+1i(sti,ati,at−i)⟵(1−α)Qti(sti,ati,at−i) +α[rt+1i(st+1i)+γmax⁡ai,a−i∈A⁡Qti(st+1i,ati,at−i)],

where *a*_*t*_^*i*^ represents the action taken by agent *i* and **a**_**t**_^−**i**^ represents the joint action taken by all the SU agents throughout the entire CR network except agent *i*. Note that *a*_*t*_^*i*^ ⋂ **a**_**t**_^−**i**^ ∈ *A*, where *A* represents joint actions by all the SU agents throughout the entire CR network. Therefore, (19) is similar to the traditional RL approach except when an action *a*_*t*_^*i*^ becomes a joint action *a*_*t*_^*i*^ ⋂ **a**_**t**_^−**i**^ (or set of actions). To take into account actions taken by the other agents **a**_**t**_^−**i**^, agent *i* updates an average *Q*-value ${\overset{-}{Q}}_{t}^{i}\left(s_{t}^{i},a_{t}^{i}\right)$, which is the average *Q*-value of agent *i* in state *s*_*t*_^*i*^ if it takes action *a*_*t*_^*i*^, while the other agents take action **a**_**t**_^−**i**^. The ${\overset{-}{Q}}_{t}^{i}\left(s_{t}^{i},a_{t}^{i}\right)$ is updated as follows:

(19) $$\begin{array}{c} {\overset{-}{Q}}_{t}^{i}\left(s_{t}^{i},a_{t}^{i}\right)=\underset{a_{-i}}{\sum }Q_{t}^{i}\left(s_{t}^{i},a_{t}^{i},a_{t}^{-i}\right)\overset{N}{\underset{j=1,j\neq i}{\prod }}\pi_{j}\left(s_{t}^{i},a_{t}^{i},a_{t}^{-i}\right), \end{array}$$

where *N* is the number of agents.

Next, ${\overset{-}{Q}}_{t}^{i}\left(s_{t}^{i},a_{t}^{i}\right)$ is applied in action selection using the Boltzmann equation (see Section 3.6). Further research can be pursued to reduce communication overheads and to enable indirect coordination among the agents.

### 4.9. Model 9: Competitive Model

Competitive model enables a SU agent to compete with its SU neighbor agents and subsequently make local decisions independently in CR networks. The competitive model enables an agent to make optimal actions in worst-case scenarios in the presence of competitor agents, which attempt to minimize the accumulated rewards of the agent. Note that the competitor agent may also possess the capability to observe, learn, and carry out the optimal actions in order to deteriorate the agents' accumulated rewards.

Wang et al. [14] propose an antijamming approach (A3) scheme called channel hopping, and it applies minimax-*Q* learning to implement the competitive model. This approach has been shown to maximize the accumulated rewards (e.g., throughput) in the presence of jamming attacks. Equipped with a limited number of transceivers, the malicious SUs aim to minimize the accumulated rewards of SU agents through constant packet transmission in a number of channels in order to prevent spectrum utilization by SU agents. The purposes of the channel hopping scheme are twofold:

- firstly, it introduces randomness in channel selection so that the malicious SUs do not jam its selected channels for data transmission;
- secondly, it selects a proper number of control and data channels in a single frequency band for control and data packet transmissions. Note that each frequency band consists of a number of channels. Due to the criticality of control channel, duplicate control packets may be transmitted in multiple channels to minimize the effects of jamming, and so a proper number of control channels are necessary.

Note that, as competitors, the malicious SUs aim to minimize the accumulated rewards of SU agents.  Table 14 shows the RL model for the scheme. Each state is comprised of four-tuple information; specifically, **s**_**k**,**t**_^**i**^ = (*P*_*k*,*t*_^*i*^, *g*_*k*,*t*_^*i*^, *N*_*C*,*k*,*t*_^*i*^, *N*_*D*,*k*,*t*_^*i*^) ∈ *S*_1_ × *S*_2_ × *S*_3_ × *S*_4_. With respect to frequency band *k*, the substate *P*_*k*,*t*_^*i*^ ∈ *S*_1_ = {0,1} represents the presence of PU activities and *g*_*k*,*t*_^*i*^ ∈ *S*_2_ = {*q*_1_, *q*_2_,…, *q*_*N*_*g*__} represents gain, while *N*_*C*,*k*,*t*_^*i*^ ∈ *S*_3_ and *N*_*D*,*k*,*t*_^*i*^ ∈ *S*_4_ represent the numbers of control and data channels that get jammed, respectively. An action *a*_*t*_^*i*^ ∈ *A* represents channel selections within a single frequency band for control and data packet transmissions purpose, and the channels may be jammed or not jammed in the previous time slot. The reward *r*_*t*+1_^*i*^(**s**_**k**,**t**+1_^**i**^, **a**_**t**_^**i**^,**a**_**t**_^**m**^) represents the gain (e.g., throughput) of using channels that are not jammed. Note that the reward *r*_*t*+1_^*i*^(**s**_**k**,**t**+1_^**i**^, **a**_**t**_^**i**^,**a**_**t**_^**m**^) is dependent on the malicious SU's (or competitor's) action **a**_**t**_^**m**^. The RL model is embedded in each SU agent.

Algorithm 7 presents the RL algorithm for the scheme. In step (b), the *Q*-function is dependent on the competitor's action **a**_**t**_^**m**^, which is thechannels chosen by the malicious SUs for jamming purpose. In step (c), the agent determines its optimal policy *π* ^*i*,∗^(**s**_**k**,**t**_^**i**^), in which the competitor is assumed to take its optimal action that minimizes the *Q*-value, and hence the term min⁡_*π*^*m*^(**s**_**k**,**t**_^**i**^)_. Nevertheless, in this worst-case scenario, the agent chooses an optimal action and hence the term argmax_*π*^*i*,∗^(**s**_**k**,**t**_^**i**^)_. In step (d), the agent updates its value function *V*(**s**_**k**,**t**_^**i**^), which is applied to update the *Q*-value in step (b) in the next time instant. Using the optimal policy *π* ^*i*,∗^(**s**_**k**,**t**_^**i**^) obtained in step (c), the agent calculates its value function *V*(**s**_**k**,**t**_^**i**^), which is an approximate of the discounted future reward. Again, the competitor is assumed to take its optimal action that minimizes the agent's *Q*-value and hence the term min⁡_*π*^*m*^(**s**_**k**,**t**_^**i**^)_.

## 5. Performance Enhancements

Table 15 shows the performance enhancements brought about by the application of the traditional and enhanced RL algorithms in various schemes in CR networks. The RL approach has been shown to achieve the following performance enhancement.

(P1) * Higher Throughput/Goodput.* Higher throughput (or goodput) indicates higher packet delivery rate, higher successful packet transmission rate, and lower packet loss rate.
(P2) * Lower End-to-End Delay/Link Delay.* Lower end-to-end delay, which is the summation of link delays along a route, indicates shorter time duration for packets to traverse from a source node to its destination node.
(P3) * Lower Level of Interference to PUs.* Lower level of interference to PUs indicates lower number of collisions with PU activities.
(P4) * Lower Number of Sensing Channels.* The lower number of sensing channels indicates lower sensing overheads (i.e., delays and energy consumption).
(P5) * Higher Overall Spectrum Utilization.* In order to increase the overall spectrum utilization, Chen et al. [24] increase channel access time, while Jiang et al. [30, 31] reduce blocking and dropping probabilities of calls, respectively.
(P6) * Lower Number of Channel Switches.* Chen et al. [24] reduce number of channel switches in order to reduce channel switching time.
(P7) * Lower Energy Consumption.* Lower energy consumption indicates longer network lifetime and number of survival nodes.
(P8) * Lower Probability of False Alarm.* Lo and Akyildiz [3] reduce false alarm, which occurs when a PU is mistakenly considered present in an available channel, in channel sensing (A2).
(P9) * Higher Probability of PU Detection.* Lo and Akyildiz [3] increase the probability of PU detection in order to reduce miss detection in channel sensing (A2). Miss detection occurs whenever a PU is mistakenly considered absent in a channel with PU activities.
(P10) * Higher Number of Channels Being Sensed Idle. *Lundén et al. [20] increase the number of channels being sensed idle, which contains more white spaces.
(P11) * Higher Accumulated Rewards.* Wang et al. [14] increase the accumulated rewards, which represent gains, such as throughput performance. Xiao et al. [38] improve SU's total payoff, which is the difference between gained rewards (or revenue) and total cost incurred.

## 6. Open Issues

This section discusses open issues that can be pursued in this research area.

### 6.1. Enhanced Exploration Approaches

While larger value of exploration probability may be necessary if the dynamicity of the operating environment is high, the opposite holds whenever the operating environment is rather stable. Generally speaking, exploration helps to increase the convergence rate of a RL scheme. Nevertheless, higher exploration rate may cause fluctuation in performance (e.g., end-to-end delay and packet loss) due to the selection of nonoptimal actions. For instance, in a dynamic channel selection scheme (A1), the performance may fluctuate due to the frequent exploration of nonoptimal channels. Similarly, in a routing scheme (A7), the performance may fluctuate due to the frequent exploration of nonoptimal routes. Further research could be pursued to investigate the possibility of achieving exploration without compromising the application performance. Additionally, further research could be pursued to investigate how to achieve an optimal trade-off between exploration and exploitation in a diverse range of operating environments. For instance, through simulation, Li [6] found that, with higher learning rate *α*  and lower temperature *τ*_*t*_, the convergence rate of the *Q*-value is faster.

### 6.2. Fully Decentralized Channel Auction Models

To the best of our knowledge, most of the existing RL-based channel auction models (see Section 4.6) have been applied in centralized CR networks, in which a centralized entity (e.g., base station) allocates white spaces to SU hosts with winning bids. The centralized entity may perform simple tasks, such as allocating white spaces to SU hosts with winning bids [16], or it may learn using RL to maximize its utility [36]. The main advantage of the centralized entity is that it simplifies the management of the auction process and the interaction among nodes. Nevertheless, it introduces challenges to implementation due to additional cost and feasibility of having a centralized entity in all scenarios. While there have been increasing efforts to enhance the performance of the RL-based auction models, further research is necessary to investigate fully decentralized RL-based auction models, which do not rely on a centralized entity, along with their requirements and challenges. For instance, by incorporating the cooperative learning feature (see Section 3.7.3) into the RL auction model, SUs can exchange auction information with PUs and other SUs in a decentralized manner, which may enable them to perform bidding decisions without the need of a centralized entity. However, this may introduce other concerns such as security and nodes' selfishness, which can be interesting directions for further research.

### 6.3. Enhancement on the Efficiency of RL Algorithm

The application of RL in various application schemes in CR networks may introduce complexities, and so the efficiency of the RL algorithm should be further improved. As an example, the collaborative model (see Section 4.8) requires explicit coordination in which the neighboring agents exchange information among themselves in order to expedite convergence to optimal joint action. This enhances the network performance at the expense of higher amount of control overhead. Hence, further research is necessary to investigate the possibility of indirect coordination. Moreover, the network performance may further improve with reduced overhead incurred by RL. As another example, while RL has been applied to address security issues in CR networks (see application (A3)), the introduction of RL into CR schemes may introduce more vulnerabilities into the system. This is because the malicious SUs or attackers may affect the operating environment or manipulate the information so that the honest SUs' knowledge is adversely affected.

### 6.4. Application of RL in New Application Schemes

The wide range of enhanced RL algorithms, including the dual *Q*-function, partial observable, actor-critic, auction, internal self-learning, collaborative, and competitive models (see Sections 4.3–4.9), can be extended to other applications in CR networks, including emerging networks such as cognitive maritime wireless ad hoc networks and cognitive radio sensor networks [40], in order to achieve context awareness and intelligence, which are the important characteristics of cognition cycle (see Section 2.2.1). For instance, the collaborative model (see Section 4.8) enables an agent to collaborate with its neighbor agents in order to make decisions on action selection, which is part of an optimal joint action. This model is suitable to be applied in most application schemes that require collaborative efforts, such as trust and reputation system [41] and cooperative communications, although the application of RL in those schemes is yet to be explored. In trust and reputation management, SUs make collaborative effort to detect malicious SUs, such that malicious SUs are assigned low trust and reputation values. Additionally, Section 3 presents new features of each component of RL, which can be applied to enhance the performance of existing RL-based applications schemes in CR networks. Further research could also be pursued to

- apply new RL approaches, such as two-layered multiagent RL model [42], to CR network applications,
- investigate RL models and algorithms applied to other kinds of networks such as cellular radio access networks [43] and sensor networks [44], which may be leveraged to provide performance enhancement in CR networks,
- apply or integrate the RL features and enhancements (e.g., state, action, and reward representations) to other learning-based approaches, such as the neural network-based approach [45].

### 6.5. Lack of Real Implementation of RL in CR Testbed

Most of the existing RL-based schemes have been evaluated using simulations, which have been shown to achieve performance enhancements. Nevertheless, to the best of our knowledge, there is lack of implementation of RL-based schemes in CR platform. Real implementation of the RL algorithms is important to validate their correctness and performance in real CR environment, which may also allow further refinements on these algorithms. To this end, further research is necessary to investigate the implementation and challenges of the RL-based scheme on CR platform.

## 7. Conclusions 

Reinforcement learning (RL) has been applied in cognitive radio (CR) networks to achieve context awareness and intelligence. Examples of schemes are dynamic channel selection, channel sensing, security enhancement mechanism, energy efficiency enhancement mechanism, channel auction mechanism, medium access control, routing, and power control mechanism. To apply the RL approach, several representations may be necessary including state and action, as well as delayed and discounted rewards. Based on the CR context, this paper presents an extensive review on the enhancements of these representations, as well as other features including *Q*-function, trade-off between exploration and exploitation, updates of learning rate, rules, and cooperative learning. Most importantly, this paper presents an extensive review on a wide range of enhanced RL algorithms in CR context. Examples of the enhanced RL models are dual *Q*-function, partial observable, actor-critic, auction, internal self-learning, and collaborative and competitive models. The enhanced algorithms provide insights on how various schemes in CR networks can be approached using RL. Performance enhancements achieved by the traditional and enhanced RL algorithms in CR networks are presented. Certainly, there is a great deal of future works in the use of RL, and we have raised open issues in this paper.

## Figures and Tables

**Algorithm 1:**
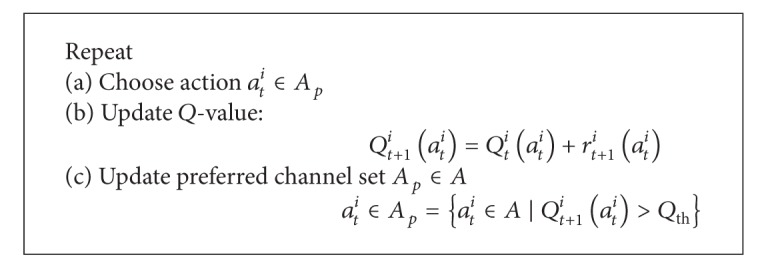
RL algorithm for joint DCS and channel sensing [10].

**Algorithm 2:**
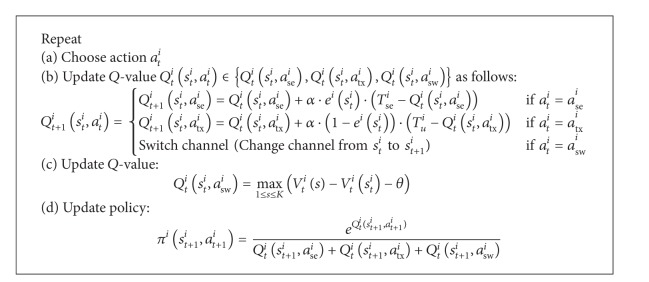
RL algorithm for joint DCS and channel sensing [11].

**Algorithm 3:**
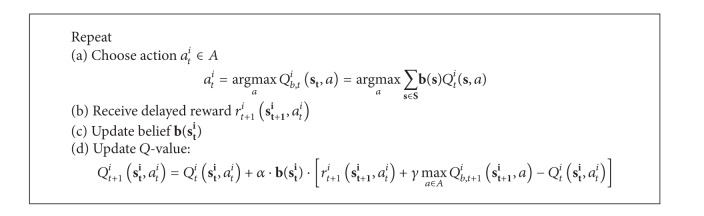
RL algorithm for joint dynamic channel selection and channel sensing [34].

**Algorithm 4:**
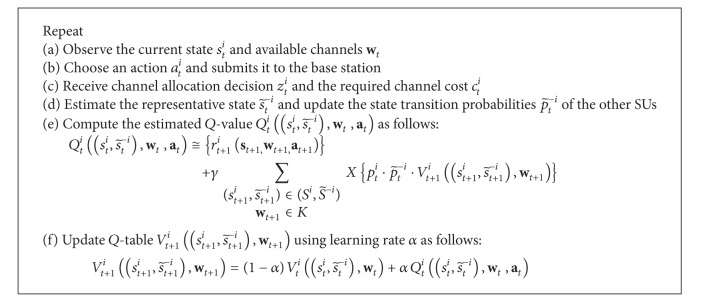
RL algorithm for the channel auction scheme [37].

**Algorithm 5:**
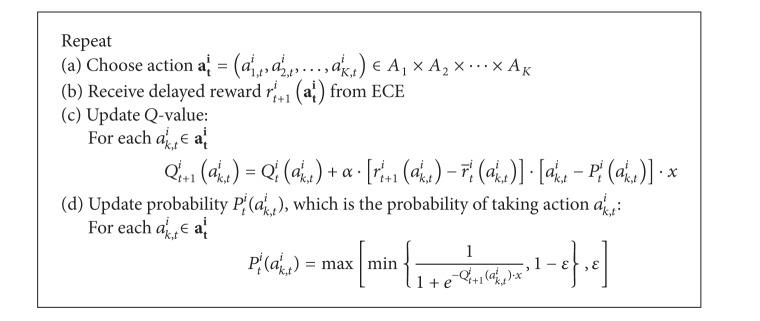
RL algorithm for RL-DCS [27].

**Algorithm 6:**
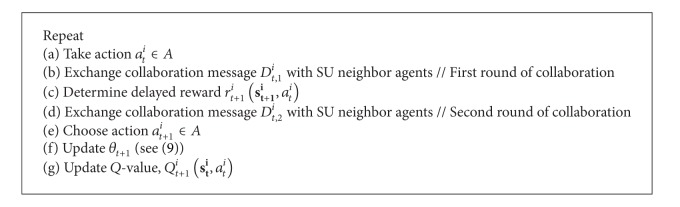
RL algorithm for the channel sensing scheme [20].

**Algorithm 7:**
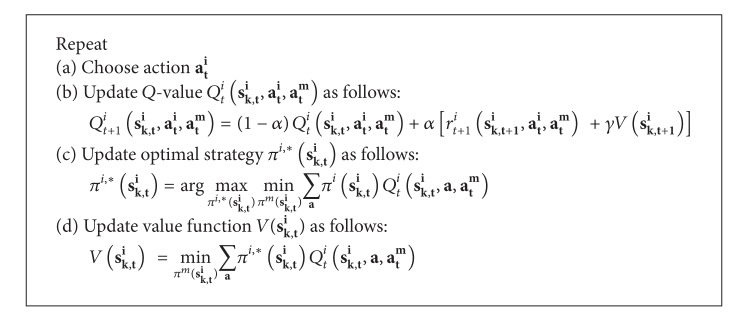
RL algorithm for the channel hopping scheme [14].

**Table 1:** RL models with direct application of the traditional RL approach for various schemes in CR networks.

References	Purpose	State	Action	Reward/cost
(A1) Dynamic channel selection (DCS)				
Tang et al. [2]	Each SU (agent) selects the operating channel with the least channel utilization level by PUs in order to improve throughput and to reduce end-to-end delay and the number of channel switches	—	Selecting an available channel for data transmission	Fixed positive/negative values to be rewarded/punished for successful/unsuccessful transmission
Li [6]	Each SU (agent) selects different operating channel with other SUs in order to reduce channel contention	—	Selecting an available channel for data transmission	Amount of successful data packet transmission
Yao and Feng [19]	SU base station (agent) selects an available channel and a power level for data transmission in order to improve its SNR. This scheme aims to increase packet delivery rate	Three-tuple information: (i) SU hosts of the SU base station, (ii) transmitting SU hosts, (iii) received power on each channel	Selecting a set of actions (see Section 3.2): (i) available channel for data transmission, (ii) transmission power level	SNR level
Li et al. [18]	Each SU link (agent) aims to maximize its individual SNR level. Note that the agent is a SU link, instead of the SU itself as seen in the other schemes	The availability of a channel for data transmission. States *s*_*k*_ = 0 and *s*_*k*_ = 1 indicate that channel *k* is idle and busy, respectively	Selecting an available channel for data transmission	SNR level, which takes into account the interference from neighboring SUs

(A2) Channel sensing				
Lo and Akyildiz [3]	Each SU (agent) (i) finds a set of neighboring SUs for cooperative channel sensing, (ii) minimizes cooperative channel sensing delay. This scheme aims to increase the probability of PU detection	A set of SU neighbor nodes that may cooperate with the SU agent to perform cooperative channel sensing	Selecting SU neighbor nodes that may cooperate with the SU agent. The SU neighbor nodes cooperate through sending their respective local sensing outcome to the SU agent	The reward (or cost) is dependent on the reporting delay, which is the time between a SU agent requesting for cooperation from a SU neighbor node and the arrival of its sensing outcome

(A4) Energy efficiency enhancement				
Zheng and Li [15]	Each SU (agent) selects a suitable action (transmit, idle, sleep, or sense channel) whenever it does not have any packets to send in order to reduce energy consumption	Four-tuple information: (i) operation mode: transmit, idle, and sleep, (ii) number of packets in the buffer, (iii) availability of PU activities, (iv) countdown timer for periodic channel sensing	Selecting an action: transmit, idle, sleep, or sense channel	Amount of energy consumption for each operation mode throughout the duration of the operation mode

(A7) Routing				
Peng et al. [4]	Each SU (agent) selects a SU neighbor node (or next hop) for data transmission to SU destination node in order to reduce end-to-end delay and energy consumption	A set of SU next hops	Selecting a SU next hop	Ratio of the residual energy of the SU next hop to energy consumption incurred by sending, receiving, encoding, and decoding data while transmitting data to the SU next hop

**Table 2:** Summary of RL models and algorithms for various schemes in CR networks.

Model	Purpose	References
Model with *γ* = 0 in *Q*-function	This model uses *γ* = 0 so that there is lack of dependency on future rewards	Li et al. [10, 17, 18]

Model with a set of *Q*-functions	This model uses a set of distinctive *Q*-functions to keep track of the *Q*-values of different actions	Di Felice et al. [11, 21]

Dual *Q*-function Model	This model updates two *Q*-functions for the next and previous states, respectively, simultaneously in order to expedite the learning process	Xia et al. [33]

Partial observable model	This model computes belief state, which is the probability of the environment operating in a particular state, in a dynamic and uncertain operating environment	Bkassiny et al. [34]

Actor-critic model	This model adjusts the delayed reward value using reward corrections in order to expedite the learning process	Vucevic et al. [13]

Auction model	This model allows agents to place bids during auctions conducted by a centralized entity so that the winning agents receive rewards	Chen and Qiu [16], Jayaweera et al. [36], Fu and van der Schaar [37], and Xiao et al. [38]

Internal self-learning model	This model enables an agent to exchange its virtualactions continuously with rewards generated by a simulated internal environment within the agent itself in order to expedite the learning process	Bernardo et al. [27]

Collaborative model	This model enables an agent to collaborate with its neighbor agents and subsequently make local decisions independently in distributed networks. A local decision is part of an optimal joint action, which is comprised of the actions taken by all the agents in a network	Lundén et al. [20] Liu et al. [39]

Competitive model	This model enables an agent to compete with its neighbor agents and subsequently make local decisions independently in worst-case scenarios in the presence of competitor agents, which attempt to minimize the accumulated rewards of the agent	Wang et al. [14]

**Table 3:** RL model for joint dynamic channel selection and channel sensing [10].

Action	*a*_*t*_^*i*^ ∈ *A*_*p*_ = {*a*_*t*_^*i*^ ∈ *A* | *Q*_*t*_^*i*^(*a*_*t*_^*i*^) > *Q*_th_}; each action represents a single channel available for data transmission

Reward	$r_{t+1}^{i}\left(a_{t}^{i}\right)\;=\{\begin{array}{cc} 1, & \text{if successful transmission}\\ -1, & \text{if unsuccessful transmission} \end{array}$

**Table 4:** RL model for joint dynamic channel selection and channel sensing [11].

State	*s*_*t*_^*i*^ ∈ *S* = {1,2,…, *K*}; each state represents an available channel

Action	*a*_*t*_^*i*^ ∈ *A* = {*a*_se_, *a*_tx_, *a*_sw_}, where action *a*_se_ senses a channel for the duration of *T*_se_^*i*^, *a*_tx_ transmits a data packet, and *a*_sw_ switches the current operating channel to another one which has the lowest best-known average transmission delay for a single-hop

Reward	*r*_*t*+1_^*i*^(*s*_*t*+1_^*i*^) represents the difference between a successful single-hop transmission delay and the maximum allowable single-hop transmission delay

**Table 5:** RL model for the routing scheme [33].

State	*s*_*t*_^*i*^ ∈ *S* = {1, 2, …, *N* − 1}; each state represents a SU destination node *n*. *N* represents the number of SUs in the entire network

Action	*a*_*t*_^*i*^ ∈ *A* = {1, 2, …, *J*}; each action represents the selection of a next-hop SU node *j*. *J* represents the number of SU node *i*'s neighbor SUs

Reward	*r*_*t*+1_^*i*^(*s*_*t*+1_^*i*^, *a*_*t*+1_^*i*^) represents the number of available common channels among nodes *i* and *j*

**Table 6:** RL model for joint DCS and channel sensing [34].

State	**s**_**t**_^**i**^ = (*s*_1,*t*_^*i*^, *s*_2,*t*_^*i*^,…, *s*_*K*,*t*_^*i*^) ∈ *S*_1_ × *S*_2_ × ⋯×*S*_*K*_; each substate *s*_*k*,*t*_^*i*^ ∈ *S*_*k*_ = {0,1} indicates an idle or busy channel; specifically, *s*_*k*,*t*_^*i*^ = 0 if PU activity does not exist in channel *k*, and *s*_*k*,*t*_^*i*^ = 1 if PU activity exists in channel *k*

Action	*a*_*t*_^*i*^ ∈ *A* = {1,2,…, *K*}; each action represents a single channel available for data transmission

Reward	$r_{t+1}^{i}\left(s_{t+1}^{i}\right)=\{\begin{array}{cc} 1, & \text{if successful transmission}\\ 0, & \text{if unsuccessful transmission because the sensed channel is busy}\\ -0.5, & \text{if unsuccessful transmission and backoff because there}\;\;\text{is collision with other SUs} \end{array}$

**Table 7:** RL model for security enhancement [13].

Action	*a*_*t*_^*i*^ ∈ *A* = {1,2,…, *N*_nbr,*i*_}; each action represents a neighboring SU chosen for channel sensing purpose, where *N*_nbr,*i*_ indicates the number of SU node *i*'s neighbor SUs

Reward	$r_{t+1}^{i}\left(a_{t}^{i}\right)\;=\{\begin{array}{cc} R, & \text{if correct sensing outcome}\\ -R, & \text{if incorrect sensing outcome} \end{array}$

**Table 8:** RL model for the channel auction scheme [16].

State	*s*_*t*_^*i*^ ∈ *S* = {0,1,…, *L*_*b*_ · *L*_*c*_}, each state represents a two-tuple information composed of buffer fullness index *L*_*b*_ and credit ratio index *L*_*c*_

Action	*a*_*t*_^*i*^ ∈ *A* = {1,2,…, *L*_*a*_}; each action represents the amount of a bid for white spaces

Reward	$r_{t+1}^{i}\left(s_{t+1}^{i}\right)\;=\{\begin{array}{cc} \text{Positive value of the amount of data sent,} & \text{if successful bid}\\ \text{Negative value of the amount of data that could have sent,} & \text{if unsuccessful bid} \end{array}$

**Table 9:** RL model for the channel auction scheme [36].

Reward	$r_{t+1}^{i}\left(a_{k,t}^{i}\right)\;=\{\begin{array}{cc} R, & \text{if successful bid}\\ -R, & \text{if unsuccessful bid} \end{array}$

**Table 10:** RL model for the channel auction scheme [37].

State	*s*_*t*_^*i*^ = (*b*_*t*_^*i*^, **p**_t_^i^) ∈ *S*; each state represents a two-tuple information composed of the fullness of the buffer state *b*_*t*_^*i*^ and channel states **p**_t_^i^ = (*p*_*t*,1_^*i*^, *p*_*t*,2_^*i*^,…, *p*_*t*,*k*_^*i*^), where *p*_*t*,*k*_^*i*^ represents the state of channel *k* in terms of SNR

Action	*a*_*t*_^*i*^ ∈ *A* = {*a*_*t*,1_^*i*^, *a*_*t*,2_^*i*^,…, *a*_*t*,*k*_^*i*^}; each action represents the amount of a bid for white spaces in channel *k*. *K* represents the number of available channels

Reward	*r*_*t*+1_^*i*^(**s**_t+1,_ **w**_t+1,_ **a**_t+1_) = *g*_*t*+1_^*i*^ + *c*_*t*+1_^*i*^ represents the sum of the number of lost packets *g*_*t*+1_^*i*^ and the channel cost *c*_*t*+1_^*i*^ that SU *i* must pay for using the channel. Note that the packet loss *g*_*t*+1_^*i*^ and channel cost *c*_*t*+1_^*i*^ depend on the global state **s**_t+1_, available channels **w**_t+1 _, and bidding actions **a**_t+1 _of all competing SUs

**Table 11:** RL model for a power control scheme [38].

Action	*a*_*t*_^*i*^ ∈ *A* = {*a*_sh_^*i*^, *a*_mh_^*i*^}, with *a*_sh_^*i*^ and *a*_mh_^*i*^ being transmitting SU *i*'s packets to the SU destination node using single-hop transmission and multiplehop relaying, respectively

Reward	*r*_*t*+1_^*i*^(*a*_*t*+1_^*i*^) represents the revenue obtained from the other SUs for relaying their packets. Higher rewards indicate higher transmission rate and transmission power of SU node *i*

**Table 12:** RL model for the DCS scheme [27].

Action	**a**_**t**_^**i**^ = (*a*_1,*t*_^*i*^, *a*_2,*t*_^*i*^,…, *a*_*K*,*t*_^*i*^) ∈ *A*_1_ × *A*_2_ × ⋯×*A*_*K*_; each subaction *a*_*k*,*t*_^*i*^ ∈ *A*_*k*_ = {0,1} represents the presence of PU activities. Specifically, *a*_*k*,*t*_^*i*^ = 0 if a PU agent cannot transmit in channel *k* and so it becomes white space, and *a*_*k*,*t*_^*i*^ = 1 if the PU agent can transmit in channel *k*.

Reward	$r_{t+1}^{i}\left(a_{t}^{i}\right)=\{\begin{array}{cc} 0, & \text{if}\;\;\hat{TH}\left(a_{t}^{i}\right)<T_{\text{th}}\\ \lambda \cdot \hat{\eta }\left(a_{t}^{i}\right)+\mu \cdot \overset{-}{W}\left(a_{t}^{i}\right), & \text{otherwise.} \end{array}$

**Table 13:** RL model for the channel sensing scheme [20].

State	**s**_**t**_^**i**^ = (*b*(*s*_1,*t*_^*i*^), *b*(*s*_2,*t*_^*i*^),…, *b*(*s*_*K*,*t*_^*i*^)) ∈ *S*_1_ × *S*_2_ × ⋯×*S*_*K*_; each substate *b*(*s*_*k*,*t*_^*i*^) ∈ *S*_*k*_ = {0,1} indicates SU *i*'s belief about channel *k*, and it has a value of 0 (busy) or 1 (idle)

Action	*a*_*t*_^*i*^ ∈ *A* = {1,2,…, *K*}; each action represents a single channel chosen for channel sensing purpose

Reward	*r*_*t*+1_^*i*^(**s**_**t**+1_^**i**^, *a*_*t*_^*i*^) represents the number of channels identified as being idle by SU node *i*

**Table 14:** RL model for the channel hopping scheme [14].

State	**s**_**k**,**t**_^**i**^ = (*P*_*k*,*t*_^*i*^, *g*_*k*,*t*_^*i*^, *N*_*C*,*k*,*t*_^*i*^, *N*_*D*,*k*,*t*_^*i*^) ∈ *S*_1_ × *S*_2_ × *S*_3_ × *S*_4_; substate *P*_*k*,*t*_^*i*^ ∈ *S*_1_ = {0,1} indicates an idle or busy channel; specifically, *P*_*k*,*t*_^*i*^ = 0 if PU activity does not exist, and *P*_*k*,*t*_^*i*^ = 1 if PU activity exists; substate *g*_*k*,*t*_^*i*^ ∈ *S*_2_ = {*q*_1_, *q*_2_,…, *q*_*N*_*g*__} represents gain, while *N*_*C*,*k*,*t*_^*i*^ ∈ *S*_3_ and *N*_*D*,*k*,*t*_^*i*^ ∈ *S*_4_ represent the numbers of control and data channels that get jammed, respectively

Action	**a**_**t**_^**i**^ = {*a*_1,*t*_^*i*^, *a*_2,*t*_^*i*^,…, *a*_*K*,*t*_^*i*^} ∈ *A*; subaction *a*_*k*,*t*_^*i*^ = (*a*_*C*_1_,*k*,*t*_^*i*^, *a*_*D*_1_,*k*,*t*_^*i*^, *a*_*C*_2_,*k*,*t*_^*i*^, *a*_*D*_2_,*k*,*t*_^*i*^), where action *a*_*C*_1_,*k*,*t*_^*i*^ (or *a*_*D*_1_,*k*,*t*_^*i*^) indicates that the agent will transmit control (or data) packets in *a*_*C*_1_,*k*,*t*_^*i*^ (or *a*_*D*_1_,*k*,*t*_^*i*^) channels uniformly selected from the previously unjammed channels, while action *a*_*C*_2_,*k*,*t*_^*i*^ (or *a*_*D*_2_,*k*,*t*_^*i*^) indicates that the agent will transmit control (or data) packets in *a*_*C*_2_,*k*,*t*_^*i*^ (or *a*_*D*_2_,*k*,*t*_^*i*^) channels uniformly selected from the previously jammed channels

Reward	*r*_*t*+1_^*i*^(**s**_**k**,**t**+1_^**i**^, **a**_**t**_^**i**^,**a**_**t**_^**m**^) represents the channel gain

**Table 15:** Performance enhancements achieved by the RL-based schemes in CR networks.

Application schemes	References	RL Models	Performance enhancements										
			(P1) Higher throughput/ goodput	(P2) Lower end-to-end delay or link delay	(P3) Lower level of interference to PUs	(P4) Lower number of sensing channels	(P5) Higher overall spectrum utilization	(P6) Lower number of channel switches	(P7) Lower energy consumption	(P8) Lower probability of false alarm	(P9) Higher probability of PU detection	(P10) Higher number of channels sensed idle	(P11) Higher accumulated rewards
(A1) Dynamic channel selection	Bkassiny et al. [34]	Partial observable			×		×						
	Tang et al. [2]	Traditional	×	×				×					
	Yao and Feng [19]	Traditional	×										
	Chen et al. [24]	Model with *γ* = 0					×	×					
	Jiang et al. [30, 31]	Model with *γ* = 0				×	×						
	Liu et al. [39]	Collaborative	×										
	Yau et al. [8, 9]	Collaborative	×					×					
	Bernardo et al. [27]	Internal self-learning	×				×						

(A2) Channel sensing	Di Felice et al. [11, 21]	Set of Q-functions	×	×	×								
	Li et al. [10]	Model with *γ* = 0	×			×							
	Lo and Akyildiz [3]	Traditional								×	×		
	Chowdhury et al. [25]	Collaborative	×	×				×			×		
	Lundén et al. [20]	Collaborative										×	

(A3) Security enhancement	Wang et al. [14]	Competitive											×
	Vucevic et al. [13]	Actor-critic								×			

(A4) Energy efficiency enhancement	Zheng and Li [15]	Traditional							×				

(A5) Auction mechanism	Jayaweera et al. [36]	Auction	×						×				
	Fu and van der Schaar [37]	Auction		×									

(A6) Medium access control	Li et al. [17]	Model with *γ* = 0	×										

(A7) Routing	Peng et al. [4]	Traditional		×					×				
	Xia et al. [33]	Dual Q-functions		×									

(A8) Power control	Xiao et al. [38]	Auction											×

## References

[1] Akyildiz IF, Lee W-Y, Vuran MC, Mohanty S (2006). NeXt generation/dynamic spectrum access/cognitive radio wireless networks: a survey. *Computer Networks*.

[2] Tang Y, Grace D, Clarke T, Wei J Multichannel non-persistent CSMA MAC schemes with reinforcement learning for cognitive radio networks.

[3] Lo BF, Akyildiz IF Reinforcement learning-based cooperative sensing in cognitive radio ad hoc networks.

[4] Peng J, Li J, Li S, Li J Multi-relay cooperative mechanism with Q-learning in cognitive radio multimedia sensor networks.

[5] Sutton RS, Barto AG (1998). *Reinforcement Learning: An Introduction*.

[6] Li H Multi-agent Q-learning of channel selection in multi-user cognitive radio systems: a two by two case.

[7] Mitola J, Maguire GQ (1999). Cognitive radio: making software radios more personal. *IEEE Personal Communications*.

[8] Yau K-LA, Komisarczuk P, Teal PD Enhancing network performance in Distributed Cognitive Radio Networks using single-agent and multi-agent Reinforcement Learning.

[9] Yau K-LA, Komisarczuk P, Teal PD Achieving context awareness and intelligence in distributed cognitive radio networks: a payoff propagation approach.

[10] Li H, Grace D, Mitchell PD Cognitive radio multiple access control for unlicensed and open spectrum with reduced spectrum sensing requirements.

[11] Di Felice M, Chowdhury KR, Meleis W, Bononi L To sense or to transmit: a learning-based spectrum management scheme for cognitive radio mesh networks.

[12] Parvin S, Hussain FK, Hussain OK, Han S, Tian B, Chang E (2012). Cognitive radio network security. *Journal of Network and Computer Applications*.

[13] Vucevic N, Akyildiz IF, Pérez-Romero J Cooperation reliability based on reinforcement learning for cognitive radio networks.

[14] Wang B, Wu Y, Liu KJR, Clancy TC (2011). An anti-jamming stochastic game for cognitive radio networks. *IEEE Journal on Selected Areas in Communications*.

[15] Zheng K, Li H Achieving energy efficiency via drowsy transmission in cognitive radio.

[16] Chen Z, Qiu RC Q-learning based bidding algorithm for spectrum auction in cognitive radio.

[17] Li H, Grace D, Mitchell PD Collision reduction in cognitive radio using multichannel 1-persistent CSMA combined with reinforcement learning.

[18] Li H, Grace D, Mitchell PD Multiple access with multi-dimensional learning for cognitive radio in open spectrum.

[19] Yao Y, Feng Z Centralized channel and power allocation for cognitive radio networks: a Q-learning solution.

[20] Lundén J, Koivunen V, Kulkarni SR, Poor HV Reinforcement learning based distributed multiagent sensing policy for cognitive radio networks.

[21] Di Felice M, Chowdhury KR, Kassler A, Bononi L Adaptive sensing scheduling and spectrum selection in cognitive wireless mesh networks.

[22] Jouini W, Ernst D, Moy C, Palicot J Upper confidence bound based decision making strategies and dynamic spectrum access.

[23] Jouini W, Moy C, Palicot J Upper confidence bound algorithm for opportunistic spectrum access with sensing errors.

[24] Chen S, Vuyyuru R, Altintas O, Wyglinski AM On optimizing vehicular dynamic spectrum access networks: automation and learning in mobile wireless environments.

[25] Chowdhury K, Doost-Mohammady R, Meleis W, Di Felice M, Bononi L Cooperation and communication in cognitive radio networks based on TV spectrum experiments.

[26] Thathachar MAL, Sastry PS (2002). Varieties of learning automata: an overview. *IEEE Transactions on Systems, Man, and Cybernetics B*.

[27] Bernardo F, Agustí R, Pérez-Romero J, Sallent O Distributed spectrum management based on reinforcement learning.

[28] Kok JR, Vlassis N (2006). Collaborative multiagent reinforcement learning by payoff propagation. *Journal of Machine Learning Research*.

[29] Reddy YB Detecting primary signals for efficient utilization of spectrum using Q-learning.

[30] Jiang T, Grace D, Liu Y (2011). Two-stage reinforcement-learning-based cognitive radio with exploration control. *IET Communications*.

[31] Jiang T, Grace D, Mitchell PD (2011). Efficient exploration in reinforcement learning-based cognitive radio spectrum sharing. *IET Communications*.

[32] Kumar S, Miikkulainen R Dual reinforcement Q-routing: an on-line adaptive routing algorithm.

[33] Xia B, Wahab MH, Yang Y, Fan Z, Sooriyabandara M Reinforcement learning based spectrum-aware routing in multi-hop cognitive radio networks.

[34] Bkassiny M, Jayaweera SK, Avery KA Distributed Reinforcement Learning based MAC protocols for autonomous cognitive secondary users.

[35] Littman ML, Cassandra AR, Kaelbling LP (1998). *Learning Policies For Partially Observable Environments: Scaling Up. Readings in Agents*.

[36] Jayaweera SK, Bkassiny M, Avery KA (2011). Asymmetric cooperative communications based spectrum leasing via auctions in cognitive radio networks. *IEEE Transactions on Wireless Communications*.

[37] Fu F, van der Schaar M (2009). Learning to compete for resources in wireless stochastic games. *IEEE Transactions on Vehicular Technology*.

[38] Xiao Y, Bi G, Niyato D (2011). Game theoretic analysis for spectrum sharing with multi-Hop relaying. *IEEE Transactions on Wireless Communications*.

[39] Liu X, Wang J, Wu Q, Yang Y Frequency allocation in dynamic environment of cognitive radio networks based on stochastic game.

[40] Zeng F, Tang Y, Pu J (2014). Multichannel broadcast based on home channel for cognitive radio sensor networks. *The Scientific World Journal*.

[41] Ling MH, Yau KLA, Poh GS (2014). Trust and reputation management in cognitive radio networks: a survey. *Security and Communication Networks*.

[42] Wang B-N, Gao Y, Chen Z-Q, Xie J-Y, Chen S-F (2007). A two-layered multi-agent reinforcement learning model and algorithm. *Journal of Network and Computer Applications*.

[43] Li R, Zhao Z, Chen X, Palicot J, Zhang H (2014). TACT: a transfer actor-critic learning framework for energy saving in cellular radio access networks. *IEEE Transactions on Wireless Communications*.

[44] Maalej M, Cherif S, Besbes H (2013). QoS and Energy aware cooperative routing protocol for wildlife monitoring wireless sensor networks. *The Scientific World Journal*.

[45] Zame W, Xu J, van der Schaar M (2014). Cooperative multi-agent learning and coordination for cognitive radio networks. *IEEE Journal on Selected Areas in Communications*.

